# Functional Relevance of the Switch of VEGF Receptors/Co-Receptors during Peritoneal Dialysis-Induced Mesothelial to Mesenchymal Transition

**DOI:** 10.1371/journal.pone.0060776

**Published:** 2013-04-09

**Authors:** María Luisa Pérez-Lozano, Pilar Sandoval, Ángela Rynne-Vidal, Abelardo Aguilera, José Antonio Jiménez-Heffernan, Patricia Albar-Vizcaíno, Pedro L. Majano, José Antonio Sánchez-Tomero, Rafael Selgas, Manuel López-Cabrera

**Affiliations:** 1 Centro de Biología Molecular-Severo Ochoa, CSIC-UAM, Cantoblanco, Madrid, Spain; 2 Unidad de Biología Molecular and Servicio de Nefrología, Hospital Universitario de la Princesa, Instituto de Investigación Sanitaria Princesa (IP), Madrid, Spain; 3 Servicio de Anatomía Patológica, Hospital Universitario de la Princesa, Instituto de Investigación Sanitaria Princesa (IP), Madrid, Spain; 4 Servicio de Nefrología. Hospital Universitario La Paz, Instituto de Investigación Sanitaria la Paz (IdiPAZ), Madrid, Spain; Cardiff University School of Medicine, United Kingdom

## Abstract

Vascular endothelial growth factor (VEGF) is up-regulated during mesothelial to mesenchymal transition (MMT) and has been associated with peritoneal membrane dysfunction in peritoneal dialysis (PD) patients. It has been shown that normal and malignant mesothelial cells (MCs) express VEGF receptors (VEGFRs) and co-receptors and that VEGF is an autocrine growth factor for mesothelioma. Hence, we evaluated the expression patterns and the functional relevance of the VEGF/VEGFRs/co-receptors axis during the mesenchymal conversion of MCs induced by peritoneal dialysis. Omentum-derived MCs treated with TGF-β1 plus IL-1β (*in vitro* MMT) and PD effluent-derived MCs with non-epithelioid phenotype (*ex vivo* MMT) showed down-regulated expression of the two main receptors Flt-1/VEGFR-1 and KDR/VEGFR-2, whereas the co-receptor neuropilin-1 (Nrp-1) was up-regulated. The expression of the Nrp-1 ligand semaphorin-3A (Sema-3A), a functional VEGF competitor, was repressed throughout the MMT process. These expression pattern changes were accompanied by a reduction of the proliferation capacity and by a parallel induction of the invasive capacity of MCs that had undergone an *in vitro* or *ex vivo* MMT. Treatment with neutralizing anti-VEGF or anti-Nrp-1 antibodies showed that these molecules played a relevant role in cellular proliferation only in naïve omentum-derived MCs. Conversely, treatment with these blocking antibodies, as well as with recombinant Sema-3A, indicated that the switched VEGF/VEGFRs/co-receptors axis drove the enhanced invasion capacity of MCs undergoing MMT. In conclusion, the expression patterns of VEGFRs and co-receptors change in MCs during MMT, which in turn would determine their behaviour in terms of proliferation and invasion in response to VEGF.

## Introduction

Peritoneal dialysis (PD) is a therapeutic option for the treatment of end-stage renal disease and is based on the use of the peritoneal membrane (PM) as a permeable barrier across which ultrafiltration and diffusion take place [1], [2]. Continuous exposure of the PM to non-physiologic PD fluids, as well as episodes of peritonitis and hemoperitoneum, may cause inflammation and injury to the PM, which progressively undergoes denudation of the mesothelial cell (MC) monolayer, submesothelial fibrosis and angiogenesis. These structural alterations may lead to the loss of the PM dialytic function [2], [3].

During long-term PD, MCs undergo a progressive loss of epithelial phenotype and acquire myofibroblast-like characteristics by a mesothelial-to-mesenchymal transition (MMT) process [2], [4], [5]. It has been demonstrated that effluent-derived MCs still retaining an epithelioid appearance *ex vivo* already show down-regulated expression of E-cadherin and cytokeratins, suggesting that the MMT of MCs starts soon after PD is initiated [4], [5]. MMT is a complex and stepwise process that is characterized by the disruption of intercellular junctions, loss of apical-basolateral polarity and acquisition of migratory and invasive properties. Cells that have undergone MMT also acquire the capacity to produce extracellular matrix components as well as inflammatory, fibrogenic and angiogenic factors [6], [7], [8], [9]. We have previously shown that effluent-derived MCs produce vascular endothelial growth factor (VEGF) spontaneously and that the MMT process of MCs is associated with strong VEGF up-regulation [10], [11]. Furthermore, we demonstrated that high levels of VEGF production by effluent MCs correlated with high transport rates in PD patients [11], [12].

VEGF is a key regulator of both physiologic and pathologic angiogenesis [13], [14]. The biological effect of this growth factor is mediated by three VEGF receptors (VEGFRs): VEGFR-1/Flt-1, VEGFR-2/KDR and VEGFR-3/Flt-4, which share similar molecular structure and are composed by seven extracellular immunoglobulin (Ig)-like domains, one transmembrane region, and an intracellular tyrosine kinase domain that is activated via ligand-triggered dimerization, leading to the induction of different signal transduction pathways [15], [16]. The activity of VEGF is also regulated by neuropilins (Nrps), a family of cell surface glycoproteins composed by two members, Nrp-1 and Nrp-2, that have about 45% amino acid identity and show conserved primary structures. These proteins are constituted by, a large extracellular domain, a single transmembrane domain and a short cytoplasmic tail. The extracellular domain contains three structural motifs: two CUB homology domains (a1/a2), two coagulation factor V/VIII homology domains (b1/b2) and a mephrin/A5-protein/protein tyrosine phosphatase µ (MAM) domain (c) [17]. The short cytoplasmic domain has no signalling motif but can interact with several proteins [18].

Nrp-1 was first characterized as a receptor for the class III semaphorins (Sema-3) in neurons mediating axon growth cone collapse [19], [20]. Subsequently, it was also described as an isoform-specific VEGF co-receptor expressed in endothelial and tumour cells, enhancing VEGF binding to VEGFR-2 and its bioactivity [21]. It has been described that Nrp-1 may also signal independently of VEGFR-2 in endothelial cells to mediate VEGF-triggered migration and adhesion [22], [23], [24], [25], [26]. The a1/a2 domains of Nrp-1 are involved in Semaphorin 3A (Sema-3A) binding, whereas VEGF binds to b1/b2 motifs [17], [27], [28]. Sema-3A and VEGF are functional competitors in their ability to bind Nrp-1 [17], [29], and promote ligand-triggered Nrp-1 internalization [30]. More recent studies revealed that Nrp-1 may also interact with other growth factors including hepatocyte growth factor (HGF) [31], fibroblast growth factor (FGF) [32], and transforming growth factor (TGF)-β1 [33], [34]. Besides neurons and endothelial cells, Nrp-1 expression has been described in many other cell types including MCs [35], [36], [37], [38], [39]. Importantly, Nrp-1 is frequently expressed by tumour cells and is involved in their malignant progression [31], [34], [40], [41], [42], [43], [44], [45], [46], [47], [48]. Nowadays, Nrps are considered potential therapeutic targets in cancer but the complex mechanisms underlying the interaction of these molecules with multiple ligands have not been fully elucidated so far. In this context, it has been described that blocking Nrp-1 function reduced tumour growth by inhibition of vascular remodelling, rendering vessels more susceptible to anti-VEGF therapy [27], [48], [49], [50], [51].

It has been demonstrated that normal and malignant MCs express VEGFRs and Nrps and that VEGF is an autocrine growth factor for mesothelioma [37]. However, the role of VEGF/VEGFRs/Nrps axis in peritoneal MCs during PD-induced MMT is still unknown. Herein, we show that MCs change the expression pattern of VEGFRs and co-receptors during MMT, which determines a switch of the VEGF effect on MCs from a proliferation response to an invasive response.

## Materials and Methods

### Ethics Statements

The protocol and informed consent were reviewed and approved by the Ethics Committee of Clinic Investigation of the ‘Hospital Universitario de la Princesa’ (Madrid, Spain). All of the patients signed the informed consent prior to the initiation of any study-related activities. This research was carried out in accordance with Good Clinical Practice guidelines, applicable regulations, as well as the ethical principles that have their origin in the Declaration of Helsinki.

### Patients

We included 51 clinically stable PD patients in this study (30 men and 21 women), ranging in age from 25 to 78 years. The causes of renal failure were diabetes (n = 16), chronic pyelonephritis (n = 12), glomerulonephritis (n = 10), nephrosclerosis (n = 9), systemic disease (n = 3) and unknown cause (n = 1). Thirty-four patients were treated with standard solution based on glucose and lactate, containing high concentration of glucose degradation products (GDPs) (Dianeal; Baxter Healthcare Corp., Deerfield, IL). Ten of these patients received one dwell per day (generally overnight) with icodextrin-containing solution (Extraneal; Baxter) and 5 received one exchange with amino acid-containing solution (Nutrineal; Baxter). Sixteen patients were treated with low-GDPs solutions buffered with lactate in 11 cases (Balance; Fresenius Medical Care, Bad Homburg, Germany) or bicarbonate in 5 cases (BicaVera; Fresenius). Finally, 1 patient received a combination of different solutions.

**Figure 1:**
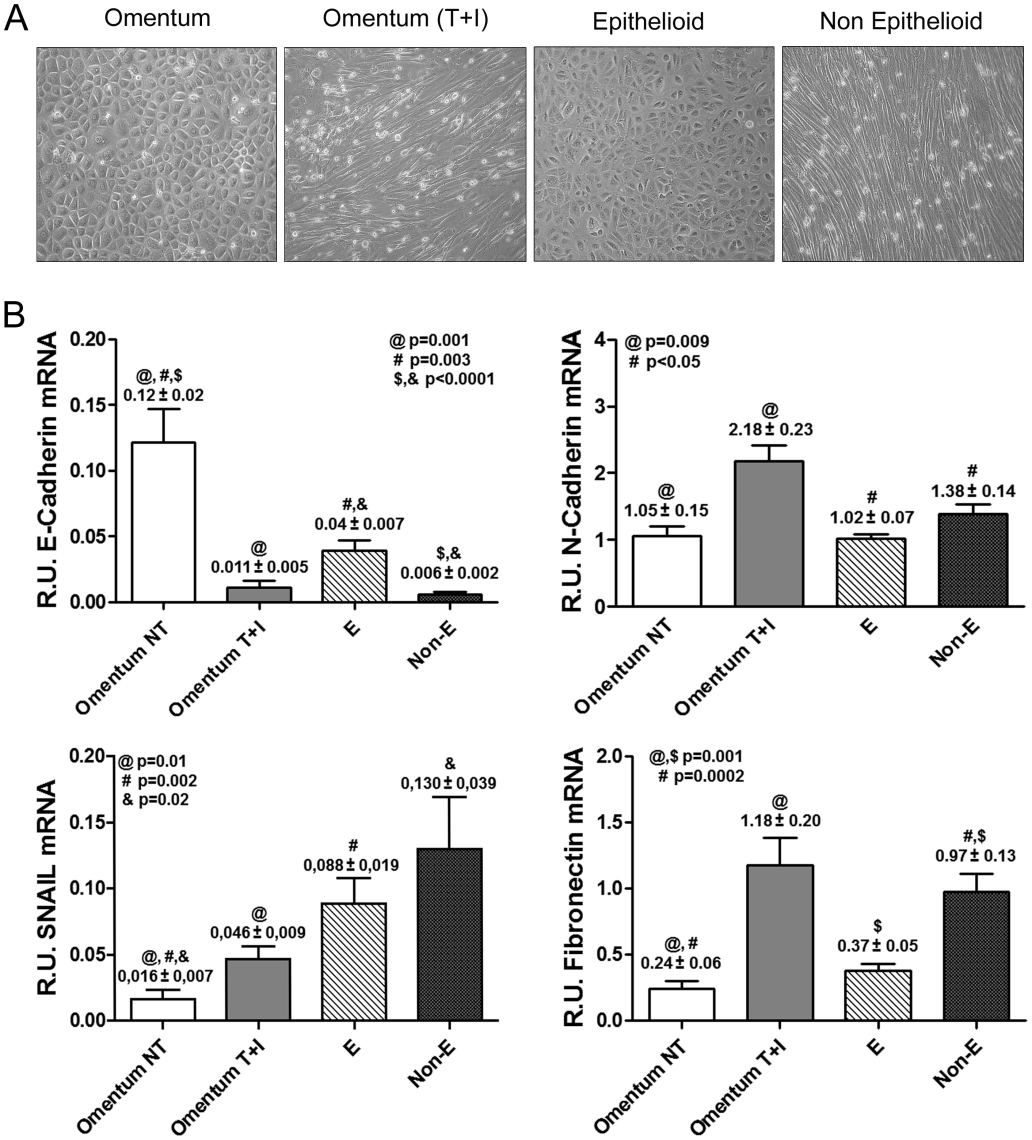
Characterization of MMT *in vitro* and *ex vivo*. (**A**) Representative pictures of omentum-derived MCs, either untreated or treated with TGF-β1 and IL-1β (MMT *in vitro*), and the two morphologies observed in confluent cultures of effluent-derived MCs: epithelioid and non-epithelioid. (**B**) Transcript levels of mesenchymal markers were analyzed by quantitative RT-PCR (n = 11 Omentum, 11 Omentum T+I, 30 E and 21 Non-E). Results show down-regulation of E-cadherin and up-regulation of snail expression during both *in vitro* and *ex vivo* MMT. The histograms also show a significant up-regulation of N-cadherin and fibronectin expression in mesenchymal MCs compared to omentum and epithelioid MCs. Data are depicted as mean value ± SE. Symbols show statistical differences between groups.

MCs from the dialysis effluents of these PD patients were classified according to morphological characteristics and the expression patterns of epithelial or mesenchymal markers (**Figure 1**) into two groups: epithelioid (E) (n = 30) and non-epithelioid (Non-E) (n = 21) [2], [11], [52], [53]. At the moment of effluent-derived mesothelial cells (MCs) sampling, 43 patients were on continuous ambulatory peritoneal dialysis and 8 were on automatic peritoneal dialysis techniques.

The baseline characteristics of the patients and the differences between the subgroups according to the phenotype of effluent-derived MCs are shown in **Table 1**. Most patients (48 of 51) received recombinant human erythropoietin during this study. The mass transfer coefficients for urea and creatinine (Urea-MTC and Cr-MTC) and the creatinine clearance (CCr) were measured using standard methods [54]. Ultrafiltration (UF) capacity was defined as a peritoneal exchange of 4 hours using 3.86% glucose (214.3 mmol/L) [55]. Peritoneal glucose load was calculated by the sum of glucose contained in each PD-fluid bag during the whole time on PD. Eleven patients showed peritonitis, and one experienced hemoperitoneum. MC cultures from effluent were taken at least 3 months after the resolution of peritonitis or hemoperitoneum episodes.

**Table 1:** Baseline characteristics of PD patients with different phenotypes of mesothelial cells.

Parameters	Studied population (n = 51)	Mesothelial Cells Phenotype		P
		Epithelioid(n = 30)	Non-Epithelioid(n = 21)	
Time on PD(months)	7.61±6.52	6.55±6.47	9.41±6.37	0.04
CCr (mL/min)	4.82±3.50	5.74±3.56	3.50±3.00	0.02
Glucose load (Kg)	22.85±28.26	24.55±33.75	20.41±18.27	NS
Urea-MTC (mL/min)	23.20±6.95	23.74±8.06	22.43±5.05	NS
Cr-MTC (mL/min)	10.85±3.13	10.39±2.72	11.50±3.60	NS
UF 3.86% (mL)*	505.90±171.58	508.80±181.64	501.76±160.40	NS
N° Peritonitis	11 from 51	2 from 11	9 from 11	0.03
VEGF (pg/mg)	20878.64±4185.47	5328.63±1005.32	43092.92±7894.40	0.0001

CCr: creatinine clearance. MTC: mass transfer coefficient. UF: ultrafiltration after a 4-hour dwell with glucose 3.86%. VEGF: vascular endothelial growth factor. Values represent mean ± SD for median clinical parameters or mean ± SE for supernatant VEGF.

### Isolation and Culture of Mesothelial Cells and Treatments

MCs from the dialysis effluents of the PD patients were isolated as previously described [56]. To standardize effluent MC harvesting, the cells were obtained from a long dwell (generally overnight) with a PD fluid containing 2.27% glucose (Dianeal; Baxter). Effluent MCs from patients treated with low-GDP liquids were isolated from a long dwell with biocompatible fluids containing 2.3% glucose and buffered with lactate (Balance, Fresenius) or with bicarbonate (BicaVera, Fresenius). Omentum-derived MCs were obtained from patients that underwent unrelated abdominal surgery as described elsewhere [56], [57]. All cells were cultured in Earle’s M199 medium, supplemented with 20% fetal-calf serum (FCS), 2% Hepes 1 M, 50 U/mL penicillin, 50 µg/mL streptomycin and 2% Biogro-2 (Biological Industries, Beit Haemek, Israel). The purity of effluent and omentum-derived MC cultures was determined by the expression of standard mesothelial markers: intercellular adhesion molecule-1, cytokeratins, and calretinine. These MC cultures were negative for von-Willebrand factor, CD31 and CD45, ruling out any contamination by endothelial cells or macrophages [56], [58], [59].

To induce MMT *in vitro*, omentum-derived MCs were treated for 72 hours with a combination of human-recombinant TGF-β1 (0.5 ng/mL) and IL-1β (2 ng/mL) (T+I) (R&D Systems, Inc, Minneapolis, MN), which has been proven to be a good *in vitro* model of MMT [4], [8], [11], [58], [59]. The cytokine doses used were in the range of those detected in PD effluents, especially during peritonitis episodes [60], and were similar to those used in previous studies [4], [8], [11], [52], [53], [58], [59], [61]. We included 31 healthy donors of omental tissue for the study, of which 11 donors were used for QT-PCR analysis and 20 for proliferation, invasion and immunofluorescence assays. Furthermore, we included 15 additional clinically stable PD patients randomly selected to obtain effluent MCs for proliferation and invasion assays, whose baseline characteristics where not considered for these experiments.

### Reagents and Antibodies

Recombinant human TGF-β1, IL-1β and Sema-3A were purchased from R&D Systems (100-B, 201-LB and 1250-S3, respectively; Minneapolis, MN,). Blocking monoclonal antibody against VEGF (MAB293, R&D Systems), and antibodies against the VEGF-binding motif or the Sema-3A-binding motif of Nrp-1 (Np-1b and Np-1a, respectively; Genentech, San Francisco, USA) [49], were used in the proliferation and invasion assays. Monoclonal antibodies IgG2a and IgG2b (Sigma-Aldrich, St Louis, MO, USA) were employed as isotype controls. Pilot studies were performed to establish optimal antibodies and recombinant protein dosages. For Western blotting anti-Nrp-1 rabbit monoclonal antibody (ab81321, Abcam, Cambridge, UK), anti-VEGFR-2 mouse monoclonal antibody (sc-316, Santa Cruz Biotechnology, Santa Cruz, CA) and anti-tubulin mouse monoclonal antibody (T-5168, Sigma-Aldrich) were used. For immunofluorescence, the same antibodies against Nrp-1 and VEGFR-2 used in Western blot were employed. Cells were also stained with anti-VEGF rabbit polyclonal antibody (ab46154, Abcam), anti-Pan cytokeratin mouse monoclonal antibody (c-1801, Sigma-Aldrich) and anti-FSP-1 (A-5114, Dako, Glostrup, Denmark). Secondary antibodies (conjugated to Alexa Fluor 568 and Alexa Fluor 488) were from Molecular Probes (Life Technologies, USA). For immunohistochemistry anti-Nrp-1 mouse monoclonal antibody (sc-5307, Santa Cruz Biotechnology) and mouse monoclonal anti-Pan cytokeratin (c-2931, Sigma-Aldrich) were employed.

### Real-time Quantitative PCR Analysis

Total RNA was extracted using TRI Reagent® (Ambion, Inc., Austin, TX) and following manufacturer’s recommendations. Complementary DNA was obtained from 2 µg of total RNA by reverse transcription (RNA PCR Core Kit; Applied Biosystems, Foster City, CA). Quantitative RT-PCR was carried out in a Light Cycler 2.0 using a SYBR Green kit (Roche Diagnostics GmbH, Mannheim, Germany) and specific primers for VEGFR-1/Flt-1, VEGFR-2/KDR and VEGFR-3/Flt-4, Nrp-1, Nrp-2, Sema-3A, E-cadherin, N-cadherin, snail, fibronectin and histone H3 (**Table S1**). Amplification values were normalized with respect to the value obtained for H3.

### Enzyme-linked Immunoassay

Albumin, VEGF and Sema-3A concentrations were determined by using standard enzyme-linked immunoassay (ELISA) kits (Abcam; R&D Systems; UscnK Life Science, USA; respectively). For analysis of VEGF levels in supernatants, the media of confluent MC cultures in the first passage was replaced with fresh media and 18 hours later supernatants were collected and stored at –80°C until their analysis. The results were normalized with total protein of cell lysates and depicted as picograms per milligrams (pg/mg). For VEGF concentration in effluents from PD patients, 15 ml of each bag of effluents were collected and stored at –80°C until their analysis. Results were represented as picograms per millilitre (pg/mL).

For Sema-3A concentration in effluents from PD patients, the dialysates were concentrated as previously described [62], using commercial concentrators (Amikon Ultra-15, Millipore). The concentration factor was defined as the albumin concentration in the concentrate divided by the albumin concentration in the original effluent sample. Results were represented as pg/mL.

### Western Blotting

MC cultures were lysed in RIPA buffer (1% sodium deoxycholate, 0.1% sodium dodecyl sulfate) plus a phosphatase and protease inhibitor cocktail (Pierce) and total protein was quantified by protein assays kit (Pierce). Equal amounts of denatured proteins (30–40 µg) from each sample were resolved by 8–10% sodium dodecyl sulfate-polyacrylamide gel electrophoresis under reducing conditions. Proteins were transferred on nitrocellulose membranes, which were then blocked with 5% nonfat milk in TBS-Tween buffer for 1 hour and incubated with specific antibodies against Nrp-1, VERGFR-2 and tubulin in 0.5% milk in TBS-Tween overnight at 4°C. These antibodies were detected with a peroxidase conjugated goat anti-mouse and donkey anti-rabbit antibodies (Pierce, Rockford, IL, USA), and then were visualized with enhanced chemiluminescence (ECL) detection kit (Pierce, Rockford, IL, USA). Blot images were acquired with GS-710 Imaging Densitometer (Bio-Rad, Hercules, CA) and analyzed with Quantify-One software (Bio-Rad).

### Confocal Microscopy and Immunofluorescence

Cells were fixed for 10 minutes in 4% paraformaldehyde and permeabilized 10 minutes in 0.1% NP-40. In all cases, 5% goat serum was applied for 20 minutes to block non-specific unions. Cells were stained for Nrp-1 (labeled with Alexa Fluor 488), VEGFR-2 (labeled for Alexa Fluor 568) and VEGF (labeled with Alexa Fluor 488) and mounted with fluorescent mounting media (Dako). Confocal images were acquired with a LSM710 Zeiss Confocal Microscope (with a 40X objective).

### Biopsy Processing and Immunohistochemical Analysis

Parietal peritoneum biopsies from PD patients were obtained from the anterior abdominal wall by surgeons during renal transplantation, insertion or removal of the PD catheter, or because of incidental abdominal conditions. Normal control samples of parietal peritoneum from non-uremic patients subjected to elective surgeries were also included in this study. Written consent was obtained from patients prior to obtaining the peritoneal samples. Tissue samples were routinely fixed in neutral-buffered 3.7% formalin (pH 7.3) during 12–24 hours. Afterwards, samples were dehydrated and embedded in paraffin to obtain sections 3–4 µm thick. Deparaffinised sections were stained with Haematoxylin-Eosin and Masson’s trichrome to analyze the histological characteristics of each specimen. Samples from 5 PD patients with evident sign of submesothelial fibrosis and 4 controls were included in this study.

For immunohistochemistry, samples were incubated with 3% hydrogen peroxide in methanol to block endogenous peroxidase activity. Antigen retrieval was performed by heating samples in citrate buffer (pH 6). Monoclonal anti-Nrp-1 (Santa Cruz Biotechnology) and anti-Pan-cytokeratin antibodies (Dako) were applied to detect the antigens by means of a dextran-polymer conjugate technique (EnVision+, Dako). The reactions were visualized by diaminobenzidine chromogen (brown) and tissue sections were finally counterstained with a nuclear haematoxylin staining (blue). Morphological characteristics were studied as previously described [4], [11].

### Proliferation Assays

For proliferation assays, 10^4^ cells/well of effluent- or omentum-derived MCs, with either epithelioid or non-epithelioid phenotypes, were seeded into 96-well plates and cultured at 37°C and 5% CO2 for 48 hours. Cells were then pulsed with [^3^H]-thymidine (1 µCi per well) for 16–18 hours and lysed with Filter Mate Cell Harvester (Perkin Elmer, Turku, Finland). Radioactivity was determined for 1 minute in a basic beta liquid scintillation counter (Perkin Elmer). For proliferation-blocking experiments, anti-VEGF (0.5, 1 or 10 µg/mL), anti-Np-1b (10 µg/mL), anti-Np-1a (10 µg/mL), and isotype controls (10 µg/mL) were added in fresh medium eight hours prior to [^3^H]-thymidine addition. Each experiment was carried out in triplicate, and at least 8 experiments were performed.

### Invasion Assays

Invasion assays were performed in a 24-well insert system (Costar, Cambridge, MA). The filters, 8 µm pore size, were pre-coated with 40 µL of 300 µg/mL solution of collagen type I (PureCol, Inamed, Fremont, Canada) allowing to gel overnight at 37°C. Then, 3×10^4^ starved MCs/well were added into the upper chamber. As a chemotactic stimulus, 5% FCS was added into the lower chamber in 600 µL of medium. Cells were allowed to invade for 24 hours at 37°C and 5% CO_2._ Transwells were then fixed in 4% formaldehyde. After removing the gel and non-invading cells from the upper face of the membrane with a cotton swab, filters were cut out and nucleus of invading cells were stained with 4′,6-diamidine-2′-phenylindole dihydrochloride (DAPI). Invading cells were counted in ten fields per sample using a fluorescence microscope (with a 40X objective). For invasion-blocking experiments, MCs were pre-incubated during 30 minutes at 4°C with recombinant Sema-3A (150 nM), anti-VEGF (0.5, 1 or 10 µg/mL), anti-Np-1b (10 µg/mL), anti-Np-1a (10 µg/mL), isotype controls (10 µg/mL), before seeding in transwell units. At least 8 experiments were carried out in duplicate.

### Statistical Analysis

Experimental data in Figures and Tables are depicted as mean ± standard error (SE). Clinical data of patients included in the study are given as mean ± standard deviation (SD). To assess the changes produced in MCs by *in vitro* MMT, induced by the treatment with TGF-β1+IL-1β, the data was analyzed using a Paired test. Comparison between data groups was performed using the non-parametric Mann Whitney rank-sum U test, Spearman regression analysis, Chi-square and 2-tail Fisher exact test. P values less than 0.05 were considered statistically significant. We used SPSS Inc, version 15 (Chicago, IL) and GraphPad Prism 4.0 (La Jolla, CA).

## Results

### Characterization of Mesothelial to Mesenchymal Transition (MMT)

For this study, we included omentum MCs from 11 donors that underwent unrelated abdominal surgery and effluent-derived MCs from 51 PD patients. To characterize the MMT process, we first identified the different morphologies of confluent MC cultures (**Figure 1A**). The effluent-derived epithelioid MCs showed cobblestone morphology similar to that of normal MCs (omentum), and effluent-derived non-epithelioid MCs showed a fibroblast-like phenotype similar to that of omentum treated with TGF-β1 and IL-1β (omentum T+I). Then, the cells were lysed for RNA extraction and we performed quantitative RT-PCR in order to study the transcript level expression of the epithelial marker E-cadherin and the mesenchymal markers N-cadherin, fibronectin and snail (**Figure 1B**). We observed a strong down-regulation of E-cadherin and up-regulation of its repressor, snail, during both *in vitro* and *ex vivo* MMT. We also observed a significant up-regulation of N-cadherin and fibronectin expression in mesenchymal MCs compared to omentum and epithelioid MCs. These data demonstrated the mesenchymal conversion of MCs.

**Figure 2:**
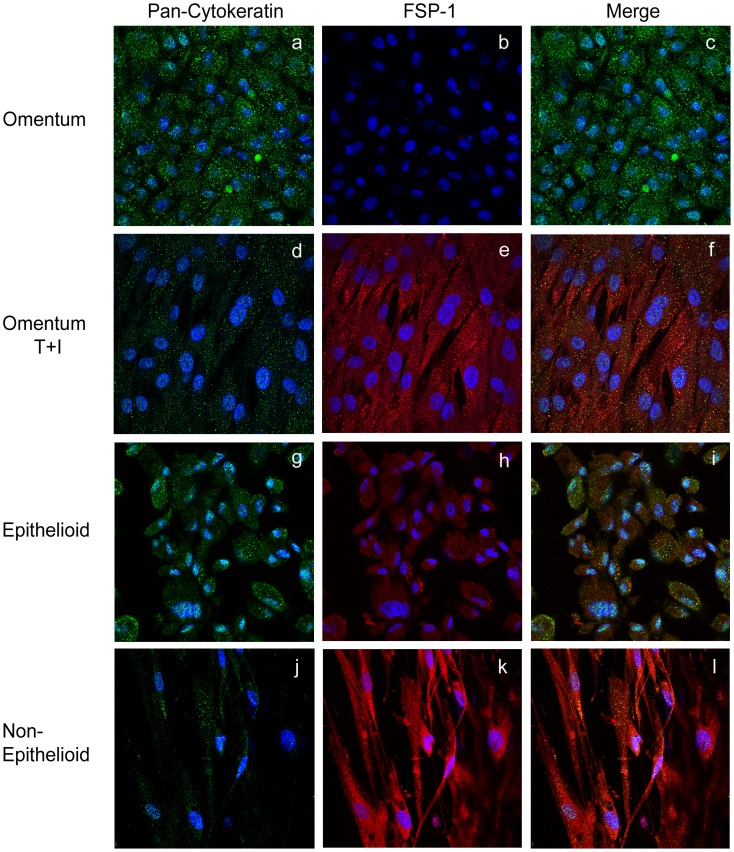
The expression of FSP-1 is up-regulated during *in vitro* and *ex vivo* MMT. The expression of FSP-1 and cytokeratin was analyzed in omentum and effluent-derived MCs by immunofluorescence using confocal microscopy. MCs were double stained for cytokeratin (green) and FSP-1 (red). Nuclei were stained with DAPI. Pictures show *in vitro* a Cyt+++/FSP1- staining for normal MCs (**a–c**), and Cyt+/FSP1++ for MCs treated with TGF-β1 and IL-1β (**d–f**). *Ex vivo*, pictures show Cyt++/FSP1+ staining for epithelioid MCs (**g–i**) and Cyt+/FSP1++ staining for non-epithelioid MCs (**j–l)** proving the mesenchymal conversion of MCs. Data are representative of 5 samples for each condition from PD patients and omentum samples included in the study.

To further characterize the MMT process, we studied the expression of fibroblast specific protein-1 (FSP-1), a mesenchymal marker, and the expression of cytokeratin (Cyt), a mesothelial marker, by immunofluorescence and confocal analysis (**Figure 2**). We observed a Cyt+++/FSP1- staining for normal MCs (**Figure 2 a–c**), and Cyt+/FSP1++ for MCs treated with TGF-β1 and IL-1β, as a consequence of *in vitro* mesenchymal conversion (**Figure 2 d–f**). In effluent-derived MCs cultured *ex vivo*, we observed a Cyt++/FSP1+ staining for epithelioid MCs (**Figure 2 g–i**). In this case cytokeratin staining showed less intensity than normal MCs and FSP-1 was also expressed, suggesting that epithelioid MCs were undergoing an early MMT. Non-epithelioid MCs showed Cyt+/FSP1+++ staining, indicating a more advanced mesenchymal transformation (**Figure 2 j–l**).

### Switch of VEGF Receptors/co-receptors during in vitro and ex vivo Mesothelial to Mesenchymal Transition

**Figure 3:**
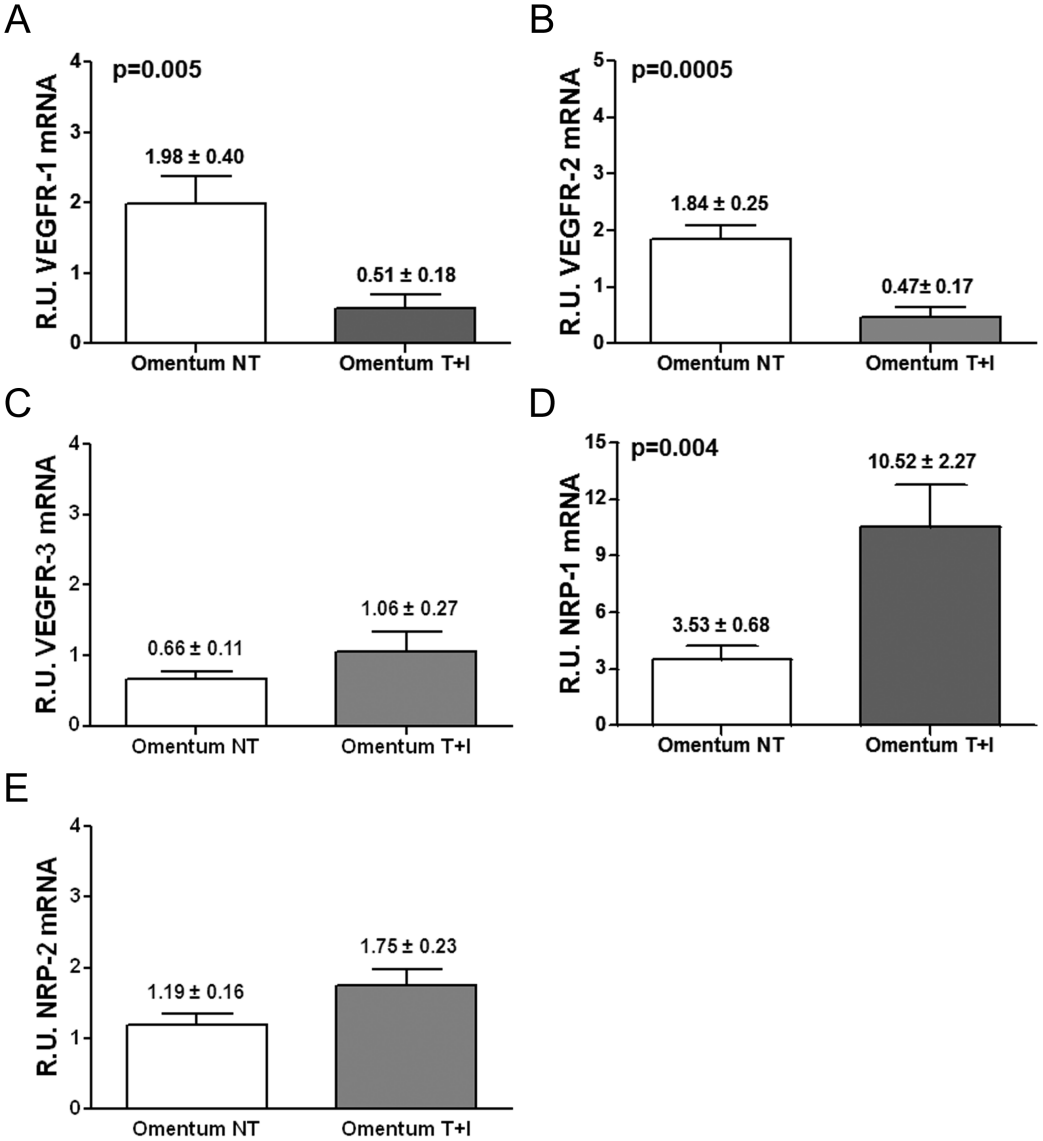
Transcript levels of VEGF receptors/co-receptors during *in vitro* MMT. mRNA levels of VEGF receptors/co-receptors were analyzed by quantitative RT-PCR. The results represent the relative mRNAs expression of VEGF receptors in omentum-derived MCs treated with TGF-β1 plus IL-1 β (Omentum T+I) compared with untreated MCs (Omentum NT). The data are depicted as mean value ± SE of omentum samples from 11 healthy donors. (**A, B and D**) The histograms show a down-regulation in expression of receptors VEGFR-1 (p = 0.005) and VEGFR-2 (p = 0.0005) and up-regulation of co-receptor Nrp-1 (p = 0.004). (**C and E**) The expression of VEGFR-3 and Nrp-2 did not show significant differences.

**Figure 4:**
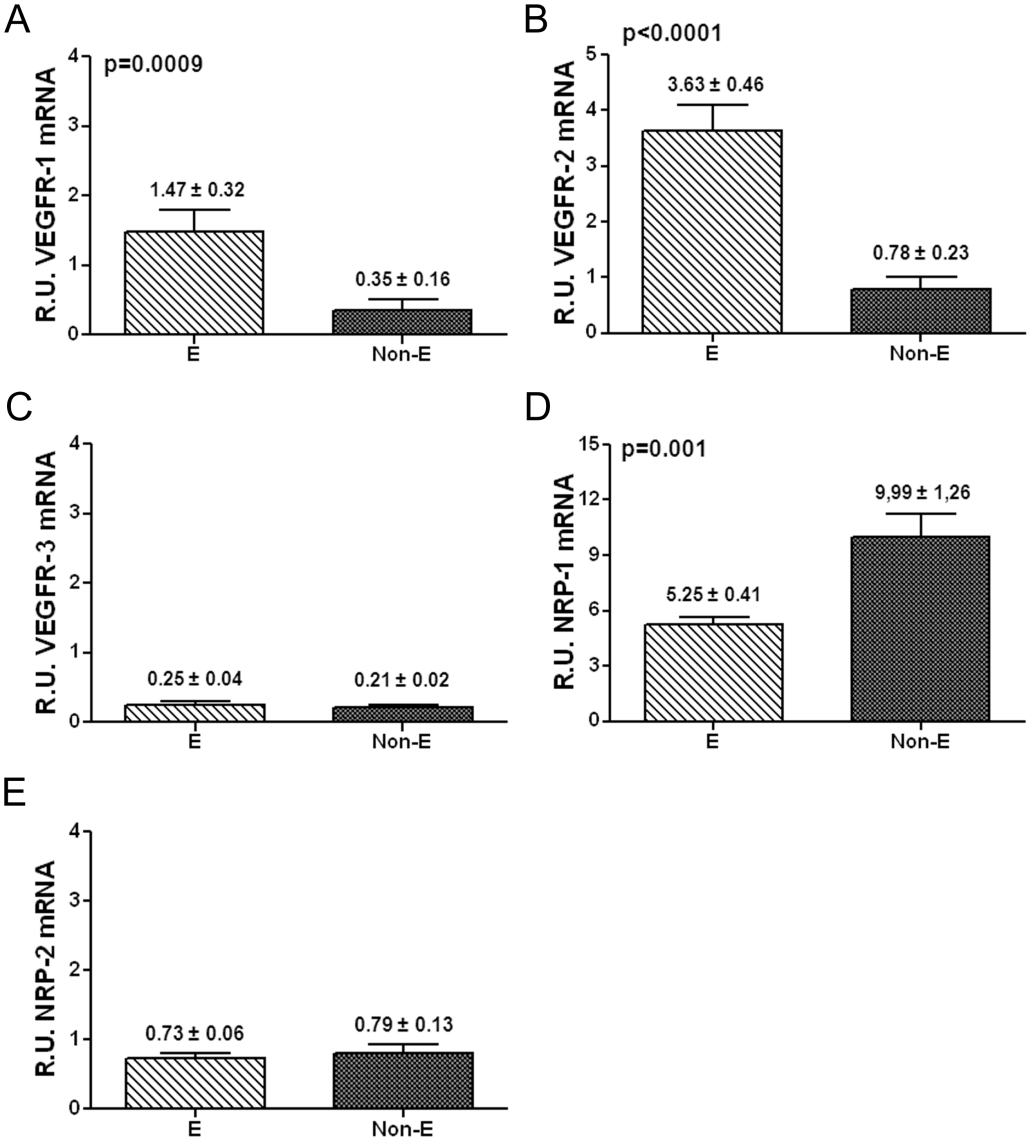
Transcript levels of VEGF receptors/co-receptors during *ex vivo* MMT. The histograms represent the relative mRNA expression of VEGF receptors/co-receptors in non-epithelioid effluent-derived MCs (Non-E, n = 21) compared with epithelioid effluent-derived MCs (E, n = 30). The data are depicted as mean value ± SE of effluent samples from 51 PD patients. (**A, B and D**) Similar results to those of *in vitro* MMT: a significant down-regulated expression of receptors VEGFR-1 (p = 0.0009) and VEGFR-2 (p<0.0001) and up-regulation of co-receptor Nrp-1 (p = 0.001). (**C and E**) The expression of VEGFR-3 and Nrp-2 did not show variation.

It has been shown that the expression of VEGFR-1/Flt-1, VEGFR-2/KDR, Nrp-1 and Nrp-2 remains unchanged during the malignant transformation of MCs (e.g. mesothelioma) [37]. However, the expression patterns of VEGFRs and co-receptors throughout the mesenchymal conversion of MCs have not been analyzed so far. Thus, we analyzed by quantitative RT-PCR the expression levels of VEGFR-1/Flt-1, VEGFR-2/KDR, VEGFR-3/Flt-4, Nrp-1 and Nrp-2 throughout *in vitro* and *ex vivo* MMT. Treatment of omentum MCs with TGF-β1 plus IL-1β (*in vitro* MMT) significantly down-regulated the expression of VEGFR-1 and VEGFR-2 (**Figures 3A and 3B**) and up-regulated the expression of the co-receptor Nrp-1 (**Figure 3D**). The expression of VEGFR-3 and Nrp-2 did not show statistical differences (**Figures 3C and 3E**). Similarly, comparison of effluent-derived MCs with different phenotypes (*ex vivo* MMT) showed significant down-regulation of VEGFR-1 and VEGFR-2 (**Figures 4A and 4B**) and up-regulation of Nrp-1 (**Figure 4D**) in non-epithelioid cells when compared with epithelioid cells. Again, the expression of VEGFR-3 and Nrp-2 did not show statistical differences (**Figures 4C and 4E**). Thus, these results evidence the switch of the expression patterns of VEGFRs and co-receptors throughout *in vitro* and *ex vivo* MMT.

These alterations are inherent features of the mesenchymal conversion of MCs and not a result of the treatment with TGF-β1 plus IL-1β. We induced a reversible *in vitro* MMT treating MCs with TGF-β1 plus IL-1β for 72 hours, and after that, we replaced the media without the cytokines for another 24 hours. We observed that MCs reverted the mRNA levels of VEGFR and co-receptors after removal of the stimuli (**Figure S1**).

### The Expression and Subcellular Localization of VEGFR-2 and Nrp-1 Proteins Change during MMT

**Figure 5:**
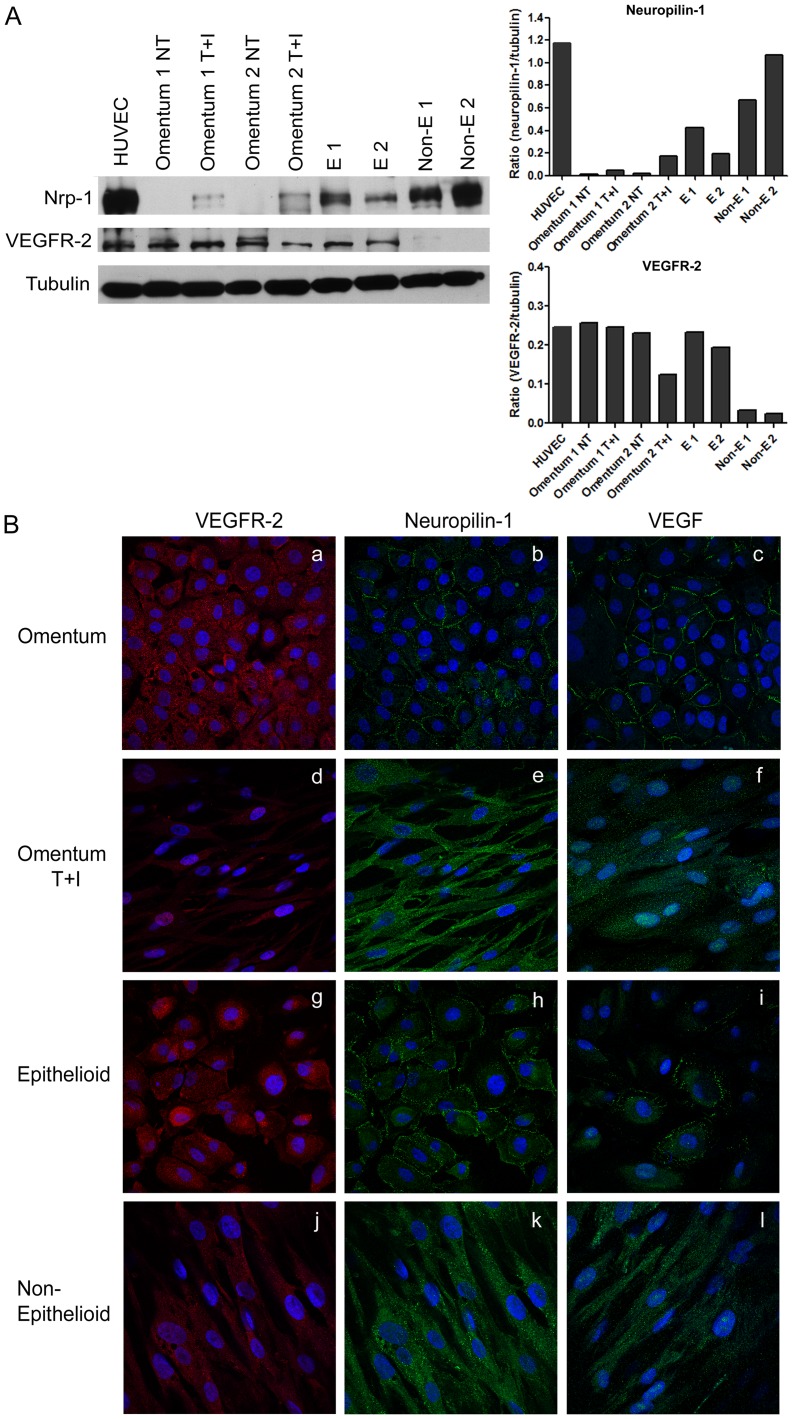
Expression levels and cellular distribution of Nrp-1 and VEGFR2 proteins during *in vitro* and *ex vivo* MMT. (**A**) Western blots show the expression levels of Nrp-1 and VEGFR-2 in total cell lysates during *in vitro* and *ex vivo* MMT. Expression of α-tubulin is employed as a loading control. Human umbilical vein endothelial cells (HUVEC) are used as a positive control. The histograms depict the quantification of Nrp-1 and VEGFR2 levels compared with α-tubulin. Data are representative of 5 samples for each condition from PD patients and omentum samples included in the study. (**B**) The expression of Nrp-1, VEGFR-2, and VEGF was analyzed by immunofluorescence microscopy in omentum and effluent-derived MCs. MCs were double stained for Nrp-1 (green) and VEGFR-2 (red), and single stained for VEGF (green). Nuclei were stained with DAPI. Nrp-1 and VEGF show a membrane distribution in omentum and epithelioid MCs (**b, c,** **h, i**)**.** During *in vitro* (**e, f**) and *ex vivo* (**k, l**) MMT both proteins change their localization and are internalized. The expression of VEGFR-2 is down-regulated but it does not show differences in localization during *in vitro* (**a, d**) and *ex vivo* (**g, j**) MMT.

We studied the expression of Nrp1 and VEGFR-2 by Western blot to confirm the data obtained by quantitative RT-PCR. Up-regulated expression of Nrp-1 and down-regulated expression of VEGFR-2 proteins were observed during both *in vitro* MMT (omentum vs. omentum T+I) and *ex vivo* MMT (epithelioid vs. non-epithelioid) **(** **Figure 5A** **)**. We also studied the cellular distribution of both proteins by immunofluorescence and we observed that Nrp-1 showed different localization depending on the cell phenotype. In epithelial-like MCs (omentum and effluent epithelioid cells), Nrp-1 was mainly localized in the membrane **(** **Figure 5B** **b, h),** whereas in mesenchymal-like MCs (omentum T+I and effluent non-epithelioid cells) the distribution was cytoplasmic, suggesting its internalization (**Figure 5B** **e, k**). In contrast, VEGFR-2 had a cytoplasmic distribution in all cell types (**Figure 5B** **a, d, g, j**). MCs stained with anti-VEGF showed a similar pattern of expression and distribution to cells stained with anti-Nrp-1 **(** **Figure 5B** **c, f, i, l)**. Moreover, pictures showed different intensity patterns for Nrp-1 and VEGFR-2 during MMT, confirming data previously observed by Western blot.

**Figure 6:**
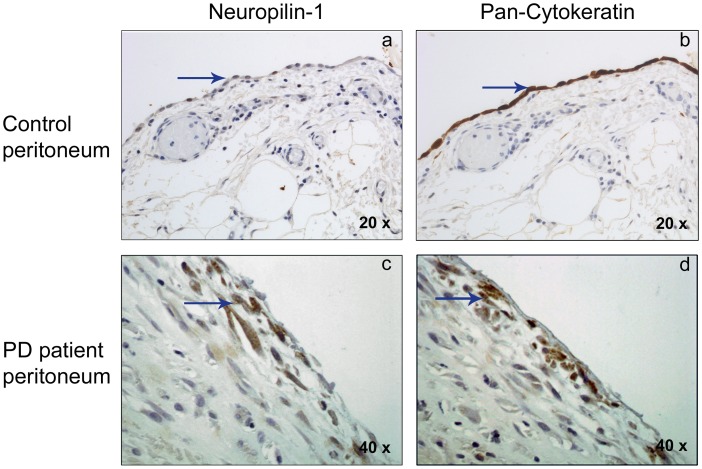
Nrp-1 immunohistochemical analysis in peritoneal human biopsies. The expression of Nrp-1 and the mesothelial marker cytokeratin was analyzed in human peritoneal specimens by immunohistochemistry. Positive cells for antibodies used (Nrp-1 and Cytokeratin) show brown staining. Nuclei are counterstained in blue. (**a, b**) Control peritoneal tissue, with a conserved mesothelial cell monolayer showing an epithelioid morphology (with a 20X objective). These cells show weak expression of Nrp-1 and a marked staining for cytokeratin (arrows). No expression of these proteins was observed in the submesothelial area (region under mesothelial monolayer) (**c, d**) Fibrotic tissue sample from PD patient showing the loss of mesothelial monolayer and invading spindle-like mesothelial cells in submesothelial area (with a 40X objective). These cells present a strong staining for Nrp-1 (**c**), and are also positive for cytokeratin (**d**) (arrows). Pictures are representative of 5 cases of PD patient samples and 4 of control samples.

To confirm *in vivo* the local up-regulation of Nrp-1 by non-epithelioid MCs, peritoneal biopsies from PD patients and controls (non-renal patients) were subjected to immunohistochemical analysis**.** Submesothelial fibroblasts from the control group did not express Nrp-1 (**Figure 6** **a**), and a weak expression of this co-receptor was confined to the surface of the MC monolayer. In contrast, peritoneal biopsies from PD patients, with evident signs of peritoneal fibrosis, showed up-regulated expression of Nrp-1 in spindle-like cells embedded in the fibrotic stroma, located mainly in the upper submesothelial area (**Figure 6** **c**). Serial sections from the same peritoneal samples showed expression of cytokeratin overlapping with Nrp-1 in the compact zone from PD patients (**Figure 6** **d**). The controls showed cytokeratin staining in the mesothelium but not in the submesothelial area (**Figure 6** **b**).

### Increase of the VEGF/Sema-3A Ratio throughout the MMT Process

**Figure 7:**
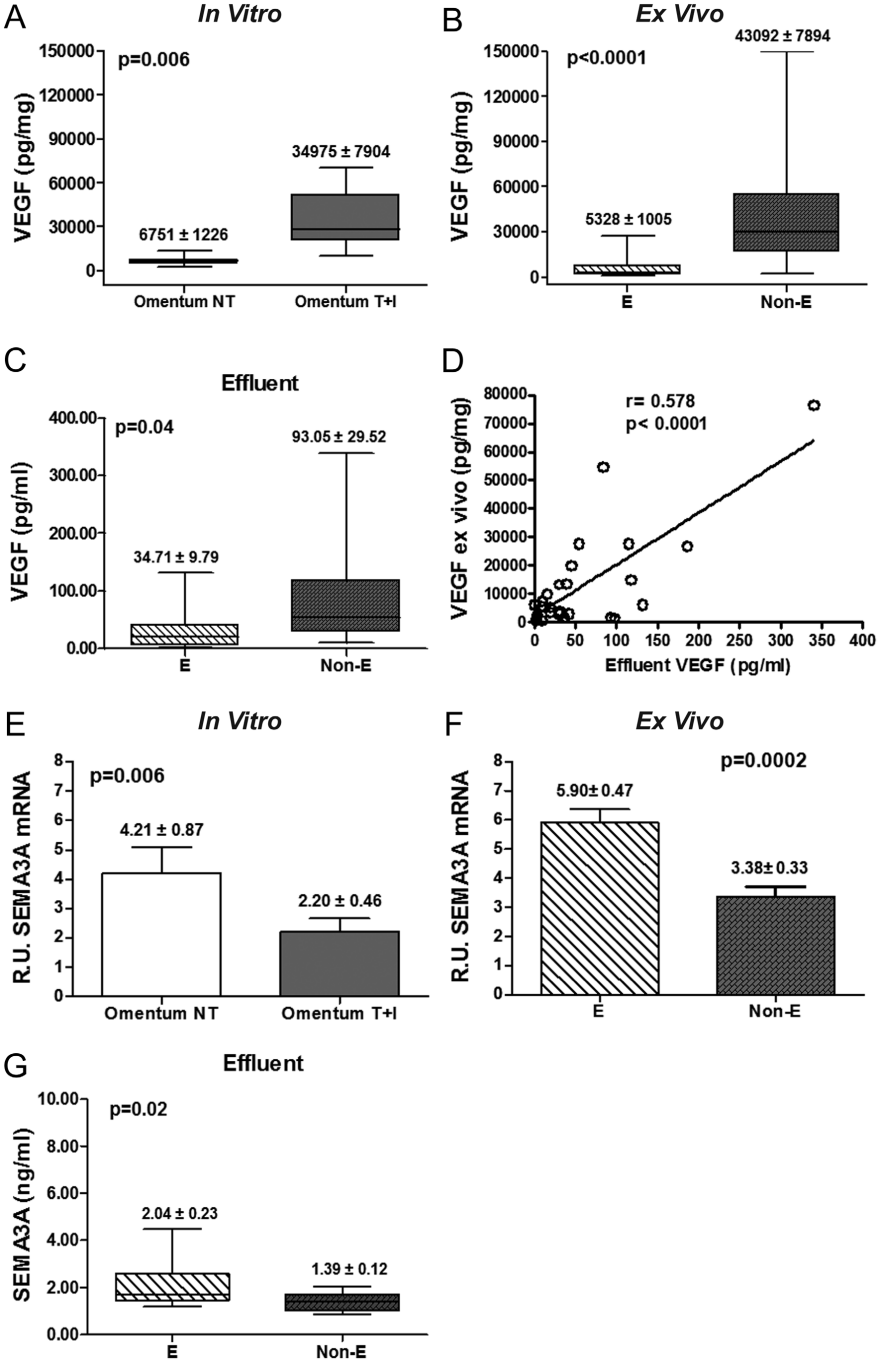
Analysis of the expression levels of VEGF and Sema3A during the MMT process. (**A and B**) The expression of VEGF in MC culture supernatants during *in vitro* (n = 11, p = 0.006) and *ex* vivo (n = 51, p<0.0001) MMT was analyzed by ELISA. (**C**) VEGF secretion in effluents of PD patients draining MCs with different phenotypes was also analyzed by ELISA (E, n = 16 and Non-E, n = 11; p = 0.04). (**D**) Correlation between VEGF levels *ex vivo* and effluents of PD patients (r = 0.578, p<0.0001) (**E and F**) Sema-3A mRNA expression, in both *in vitro* (p = 0.006) and *ex vivo* (p = 0.0002) MMT, was analyzed by quantitative RT-PCR. (**G**) Sema-3A secretion in PD effluents (n = 16 E and 11 Non-E; p = 0.02). Box Plots represent 25% and 75% percentiles, median, minimum and maximum values. Numbers above boxes and histograms depict mean ± SE.

A previous study from our group described that MMT is associated with strong up-regulation of VEGF expression [10]. In the present study, we confirmed the induction of VEGF secretion to culture supernatants in omentum MCs treated with TGF-β1 plus IL-1β (*in vitro* MMT) and in effluent MCs with non-epithelioid phenotype (*ex vivo* MMT) (**Figures 7A and 7B**). Furthermore, we determined the levels of VEGF secreted in the dialysates of PD patients and we observed higher levels of VEGF in effluents of patients that drained non-epithelioid MCs than in effluents with epithelioid MCs (**Figure 7C**). Moreover, we found a significant correlation between VEGF levels secreted *ex vivo* and VEGF levels secreted in effluents (**Figure 7D**).

On the other hand, it has been shown that Sema-3A might act as a functional competitor of VEGF-driven cell responses through the competition for Nrp-1 binding [37], [63]. Thus, we measured Sema-3A mRNA expression throughout the MMT process. Significant down-regulation of Sema-3A-encoding transcript took place in both *in vitro* and *ex vivo* MMT (**Figures 7E and 7F**). We also analyzed the concentration of Sema-3A in the effluents. We found significant lower levels of this protein in effluents that drained non-epithelioid MCs. (**Figure 7G**). In addition, the expression of VEGF and Sema-3A showed a significant negative correlation (**Figure S2**). These data demonstrate that the VEGF/Sema-3A ratio increases throughout the MMT process.

### The Change of the VEGF/VEGFRs/Nrp Axis is Associated with Peritoneal Functional Decline in PD Patients

We analyzed the baseline characteristics of PD patients included in this study and the differences between subgroups according to the phenotype of effluent-derived MCs (**Table 1**). Significant differences in the time on PD, the CCr and *ex vivo* production of VEGF were found between epithelioid and non-epithelioid subgroups of MCs. Nine of the 11 patients who experienced peritonitis, and the patient that suffered hemoperitoneum, drained non-epithelioid MCs in their effluents (p = 0.03). These data suggest that MCs with non-epithelioid phenotype appear at early stages of peritoneal membrane deterioration, even before ultra-filtration failure has been established.

An elevated mass transport coefficient for creatinine (Cr-MTC) is a clinical marker for peritoneal functional decline, which is related to the augmented vessel number and is associated with increased production of VEGF by MCs [10]. Thus, we subdivided the PD patients into 2 groups according to peritoneal transport characteristics: Cr-MTC <11 mL/min (low and low-average transporters) and Cr-MTC ≥11 mL/min (high and high-average transporters) [7], [10]. As expected, the high transporter group showed a significant increase of urea-MTC and glucose load and a significant decrease of ultra-filtration (**Table 2**). In addition, Cr-MTC seemed to be associated with effluent MC phenotype; 30% of patients (9 of 30) with epithelioid MCs in their effluent showed Cr-MTC ≥11, whereas 62% of patients (13 of 21 patients) with non-epithelioid MCs showed Cr-MTC ≥11 (two tails Fischer's exact test; r = 0.395, p = 0.04) (**Table S2**). In agreement with our previous results [10], effluent-derived MCs from high transporters produced significantly higher *ex vivo* amounts of VEGF than MCs from low transporters (**Table 2**). In addition, we observed a significant correlation between the high peritoneal transport status and the down-regulation of VEGFR-2 and Sema-3A mRNAs *ex vivo*. The high transporter group showed augmented expression of Nrp-1-encoding mRNA, although it did not reach statistical significance (**Table 2**). Of note, the increased expression of Nrp-1 had a significant correlation with the time on PD **(**p = 0.001). These data indicated that the peritoneal functional decline was associated to changes of MC phenotype and to changes of VEGF/VEGFRs/Nrp axis.

**Table 2:** Differences between low and high peritoneal transporters.

	Cr-MTC <11mL/min (n = 29)	Cr-MTC ≥11mL/min(n = 22)	P
Urea-MTC (mL/min)	21.23±3.53	25.80±9.30	0.02
Albumin (g/dL)	3.64±0.46	3.46±0.36	0.04
UF rate (3.86%)	574.66±180.61	415.27±107.10	0.0001
Glucose load (Kg)	20.99±33.56	25.29±19.72	0.01
N° peritonitis	4 from 11	7 from 11	NS
VEGF (pg/mg)	14319.43±4242.77	29524.86±7667.62	0.01
VEGFR-1 (R.U)	2.00±0.73	0.93±0.36	NS
VEGFR-2 (R.U)	3.73±0.65	1.52±0.35	0.005
VEGFR-3 (R.U)	0.22±0.02	0.24±0.05	NS
Nrp-1 (R.U)	6.21±0.64	8.29±1.24	0.14 (NS)
Nrp-2 (R.U)	0.74±0.08	0.75±0.10	NS
Sema-3A (R.U)	5.59±0.48	4.06±0.43	0.025

Statistic differences between the groups of low, low-average (Cr-MTC <11 mL/min, range 4.3–10.9) and high, high-average (Cr-MTC ≥11 mL/min, range 11.3–18.3) peritoneal transporters. R.U. mRNA relative units. Values represent mean ± SD for median clinical parameters or mean ± SE for experimental data.

### Effects of VEGF- and Nrp-1-blocking Antibodies on MC Proliferation and Invasion

We hypothesized that VEGF might establish loops in MCs to control key processes, such as proliferation and/or invasion. Our results suggest that the pair VEGF/Nrp-1 appear to gain relevance throughout MMT, given that both are up-regulated during the whole process. Of note, the up-regulated expression of VEGF and Nrp-1 showed a strong correlation (**Figure S2**). Thus, we investigated whether these two molecules played a major role in proliferation and/or invasion of MCs during the MMT process.

**Figure 8:**
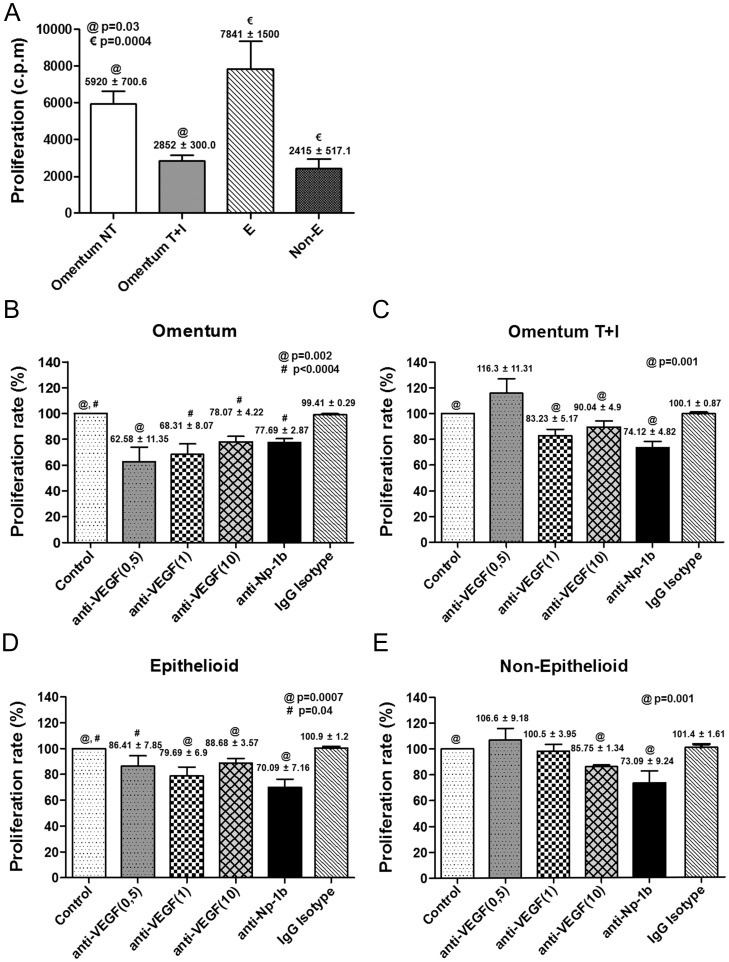
Treatment with anti-VEGF and anti-Np1b blocking antibodies interferes with the proliferation of mesothelial cells. (**A**) The proliferation capacity of MCs throughout *in vitro* and *ex vivo* MMT was analyzed by the incorporation of [3H]-thymidine (n = 20). Bar graphic depicts the radioactivity emitted (c.p.m.) in each condition. Data are depicted as mean value ± SE. Symbols show statistical differences between groups. (**B and E**) Effect of anti-VEGF (n = 10) and anti-Np1b antibodies (n = 8), or IgG isotype control antibody on the proliferation capacity of omentum-derived MCs, either untreated (**B**) or treated with TGF-β1 plus IL-1β (**C**); and on the proliferation capacity of effluent-derived MCs with epithelioid (**D**) or non-epithelioid phenotype (**E**). Bar graphics represent proliferation percentage of treatments over control cells. Data are depicted as mean value ± SE. Symbols show statistical differences between groups.

Analysis of the proliferation capacity showed that throughout *in vitro* and *ex vivo* MMT there was growth arrest of MCs (**Figure 8A**). Blocking of endogenously produced VEGF with a specific antibody resulted in significant inhibition of the proliferation of omentum MCs (**Figure 8B**). In contrast, the lower proliferation capacity of mesenchymal-like MCs (T+I-treated omentum MCs and Non-E effluent MCs) was not affected, or only marginally affected, by treatment with anti-VEGF (**Figures 8C and 8E**). Surprisingly, the proliferation capacity of effluent MCs with epithelioid phenotype, which was similar to that of omentum MCs, was only slightly affected by the VEGF-blocking antibody (**Figure 8D**). These data suggest that endogenous VEGF only plays a role on cellular proliferation in naïve epithelial-like MCs, but not in MCs at any stage, early or late, of the mesenchymal conversion. Treatment with blocking anti-Np-1b antibody inhibited cellular proliferation to a similar extent (25 to 30%) in MCs with both epithelial and mesenchymal phenotypes **(** **Figures 8B to 8E**), suggesting that Nrp-1 mediated not only the VEGF-dependent but also the VEGF-independent proliferation response. In this context, anti-Np-1a antibody, which did not interfere in VEGF-Nrp-1 interaction, also partially inhibited the proliferation of mesenchymal-like MCs (data not shown).

**Figure 9:**
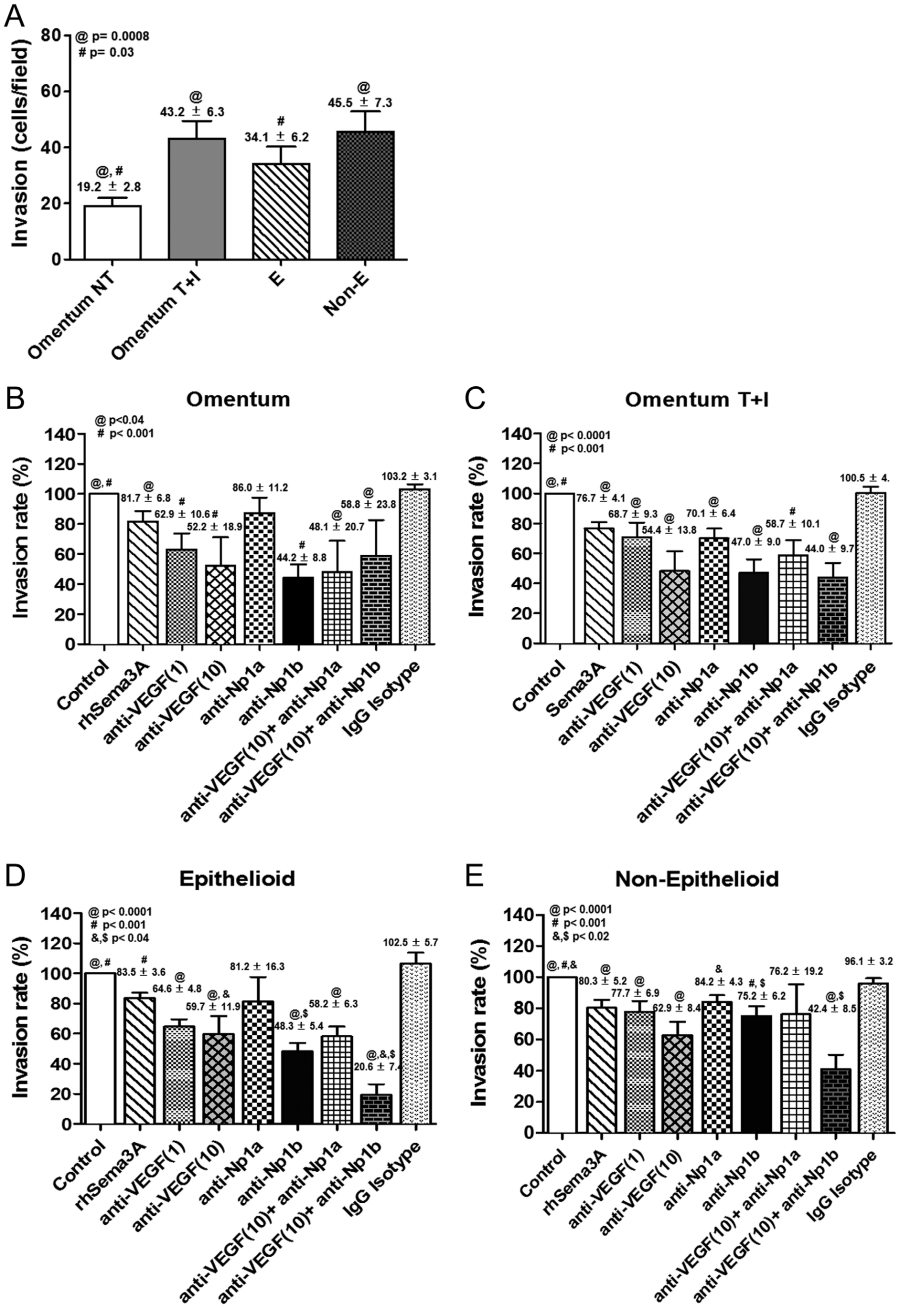
Effects of anti-VEGF and anti-Np1b blocking antibodies on the invasion capacity of mesothelial cells. (**A**) The MC invasion capacity throughout *in vitro* and *ex vivo* MMT was analyzed by counting invading cells using a fluorescence microscope (n = 20). Data are depicted as mean value ± SE. Symbols show statistical differences between groups. (**B and E**) Effect of rhSema3A (n = 15), anti-VEGF (n = 10), anti-Np1a (n = 8), anti-Np1b (n = 8) and IgG Isotype antibodies on the invasion capacity of omentum-derived MCs, either untreated (**B**) or treated with TGF-β1 plus IL-1β (**C**); and on the invasion capacity of effluent-derived MCs with epithelioid (**D**) or non-epithelioid (**E**) phenotype. Bar graphics represent invasion percentage of treatments over control cells. Numbers above histograms depict mean ± SE. Symbols show statistical differences between groups.

Invasion assays using omentum MCs demonstrated that *in vitro* MMT enhanced the invasion capacity. On the other hand, effluent-derived MCs with both epithelioid and non-epithelioid phenotypes showed increased invasion capacity when compared with omentum-derived MCs (**Figure 9A**). Treatment of omentum MCs with anti-VEGF and anti-Np-1b antibodies significantly inhibited the invasion capacity, and co-treatment with both antibodies had no additive effect (**Figure 9B**). Treatment of MCs that were at any stage of the MMT process including epithelioid MCs from effluents with anti-VEGF and anti-Np-1b antibodies showed also an important significant inhibition of the invasion capacity, and co-treatment with both antibodies had co-operative effects (**Figures 9C to 9E** **)**. Treatment of omentum- and effluent-derived MCs with anti-Np-1a, which did not block the VEGF-Nrp-1 interaction [49], showed partial (**Figures 9B and 9D** **)** or no statistically significant effects on cellular invasion (**Figures 9C and 9E** **)**. In addition, treatment of MCs with recombinant Sema-3A, which has been shown to compete with VEGF for binding to Nrp-1, resulted in a statistically significant diminished invasion capacity, reinforcing the notion that the interaction of VEGF with Nrp-1 participated, at least partially, in MC invasion (**Figures 9B to 9E** **)**.

## Discussion

The presence of MCs that have undergone a MMT in the effluent and in the peritoneal tissue of PD patients was first demonstrated in a landmark paper published in 2003 [4]. MCs with mesenchymal phenotype acquire the capacity to synthesize extracellular matrix components as well as pro-inflammatory and pro-angiogenic factors [2]. During the last few years, emerging evidence has suggested that the mesenchymal conversion of MCs is an important event for structural and functional peritoneal deterioration [7], [10], [64], [65]. In this context, we have previously shown that during the MMT process there is a strong up-regulation of VEGF and that high levels of VEGF production by effluent-derived MCs correlated with high transport rates in PD patients [10]. In this study we show that, not only VEGF expression levels, but also the whole VEGF/VEGFRs/co-receptors axis, is associated with high peritoneal transport status.

**Figure 10:**
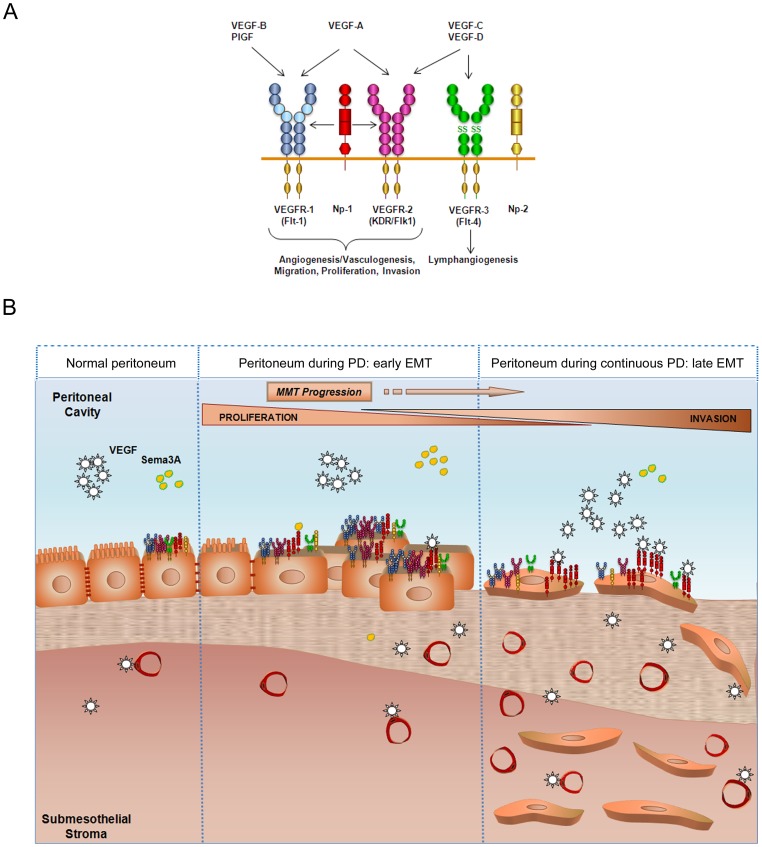
Proposed model of the role of VEGF receptors and co-receptors during MMT induced by peritoneal dialysis. (**A**) General scheme of VEGF receptors and co-receptors and the processes in which they are involved. (**B**) A normal peritoneum shows baseline VEGF receptors and co-receptors expression, and normal VEGF and Sema-3A levels are secreted at the peritoneal cavity. During continuous peritoneal dialysis, MMT takes place in the peritoneum. Denudation of MC monolayer, submesothelial fibrosis, and augmented vessel number begin to appear as visible signs as a result of the MMT. In addition, MCs change the expression pattern of VEGFRs and co-receptors. At an initial stage, a higher proliferation rate of MCs takes place, perhaps in order to repair the damage in the monolayer. However, MCs could fail to repair the peritoneal damage and MMT progress. As a result of MMT progression, MCs increase secreted VEGF levels while its receptors are down-regulated and Nrp-1 co-receptor increases at late stages of the MMT. The binding of VEGF to Nrp-1 would induce MC invasion to the submesothelial stroma. Therefore, MMT would determine MC behaviour in terms of proliferation and invasion in response to VEGF.

Peritoneal fibrosis is the most common structural change observed in PD patients, and it has been considered the main cause for the progressive functional decline of the peritoneum. However, in parallel with fibrosis, the peritoneum may also experiment an increase in capillary number (angiogenesis) in response to PD. Some reports have evidenced that enhancement of peritoneal vasculature and vessel permeability appear to be responsible for an increase in solute transport across the peritoneal membrane [66], [67]. It is generally accepted that local production of VEGF by MCs during PD may exert paracrine effects on endothelial cells to induce peritoneal angiogenesis and functional decline [10], [62], [68], [69]. Furthermore, VEGF has been shown to inhibit endothelial to mesenchymal transition [70], [71]. Therefore, it is conceivable that VEGF secreted by mesothelial cells could exert its function on myofibroblasts with an endothelial origin localized in the compact zone [5], inhibiting the mesenchymal phenotype by a mesenchymal to endothelial transition and, consequently, inducing angiogenesis. However, the possible autocrine effects of VEGF on MCs throughout the MMT process have been overlooked. The expression of VEGFRs and Nrps by MCs has been previously described [37]. Furthermore, it has been shown that the expression pattern of VEGFRs and co-receptors remains unaltered during the development of mesotheliomas [37]. In contrast, herein we show that throughout the mesenchymal conversion of MCs, the expression of the receptors VEGFR-1 and VEGFR-2 is down-regulated, and the expression of the co-receptor Nrp-1 is induced. In addition, we demonstrate that the ratio VEGF/Sema-3A increases sharply during the MMT process. It is intriguing that the main receptor VEGFR2, involved in VEGF-mediated proliferation, is repressed during MMT while the expression of VEGF is strongly induced. It is also noteworthy that the expression of Nrp-1, implicated in multiple cellular functions including invasion, is induced during MMT in parallel with VEGF. In this context, the expression of VEGF shows a significant negative correlation with VEGFR2 and a significant positive correlation with Nrp-1 (**Figure S2**). Interestingly, VEGF and Nrp1 have a very similar distribution in MCs and during MMT, when both proteins seem to internalize. However, VEGFR2 has a different distribution. Thus, it is tempting to speculate that VEGF might have divergent autocrine functions (e.g. proliferation vs. invasion) on MCs with either epithelial or mesenchymal phenotype **(** **Figure 10**).

In this study, we demonstrate that MCs that have undergone an *in vitro* or *ex vivo* MMT proliferate less than epithelial-like MCs. These data are in agreement with previous results that demonstrated that during EMT, there is an arrest of the cell cycle and the cells acquire resistance to death [72]. Furthermore, we show that blockade of endogenous VEGF inhibits the proliferation of omentum MCs. Surprisingly, the proliferation capacity of epithelioid MCs from effluents is only marginally affected by VEGF-blocking antibody, in spite of producing similar amounts of VEGF and expressing even greater levels of VEGFR2 than omentum MCs. It is noteworthy that effluent MCs, still retaining an epithelioid appearance, already show down-regulated expression of E-cadherin and induction of snail, suggesting that these cells are already in early stages of the MMT process [4] (**Figure 1**). Thus, our results indicate that endogenous VEGF plays a key role in cellular proliferation in naïve epithelial-like MCs, but not in MCs undergoing the MMT process. On the contrary, we demonstrate that MCs at any stage of the mesenchymal conversion acquire an increased invasion capacity compared with naïve epithelial-like MCs. In addition, we show that the enhanced invasion can be partially inhibited by treatment with anti-VEGF or anti-Np-1b antibodies, and is almost completely abrogated by a combination of both antibodies. These results strongly suggest that the interaction of VEGF with Nrp-1 may have a role in MC invasion **(** **Figure 10** **)**.

Perhaps the most important aspect of the identification of the MMT as a key event in peritoneal deterioration is that this process can be modulated *in vivo* with a number of endogenous factors and pharmaceutical agents [2]. In recent works, therapeutic strategies were designed either to prevent or reverse the MMT process itself or to reduce the MMT-inducing stimuli such as inflammation and AGEs [7], [11], [12], [73]–[75]. It is important to point out that MMT is a physiologic process necessary for wound healing during PD-induced aggression of the peritoneum; therefore, it is plausible that chronic blockade of MMT would result in inefficient tissue repair. Thus, alternative therapeutic approaches should be addressed to treat the consequences of the MMT, instead of the MMT process itself. One of such consequences of MMT is the increased cellular invasion. The identification of VEGF and Nrp-1 as important molecules controlling MC invasion might contribute to the design of therapeutic approaches to avoid the accumulation of MC-derived myofibroblasts in the submesothelial compact zone **(** **Figure 10** **)**.

## Supporting Information

Figure S1 **Effect on VEGF receptors and co-receptors after TGF-β1 and IL-1β removal. (A–C)** mRNA levels of VEGF receptors/co-receptors were analyzed by quantitative RT-PCR. The results represent the fold induction of mRNA expression of VEGF receptors in omentum-derived MCs treated with TGF-β1 plus IL-1 β (T+I 72 h) and omentum-derived MCs after T+I withdrawal (T+I 72 h/24 h wo) compared with untreated MCs (NT). **(D–F)** mRNA levels of mesenchymal markers were analyzed by quantitative RT-PCR. Histograms represent the expression of E-cadherin, collagen I and fibronectin after the treatment with T+I (T+I 72 h) and after T+I withdrawal (T+I 72 h/24 h wo) compared to non treated omentum-derived MCs (NT).
(TIF)

Figure S2 **Correlations between secreted levels of VEGF and mRNA levels in effluent-derived MCs. (A)** Negative correlation between VEGF levels and VEGFR-2 mRNA expression levels (p = 0.004). **(B)** Negative correlation between VEGF levels and Sema-3A mRNA expression (p = 0.04). **(C)** Positive correlation between secreted VEGF and Nrp1 mRNA expression levels (p<0.0001). Data are depicted as mean value ± SE. Symbols show statistical differences between groups.
(TIF)

Table S1 **Oligonucleotides Sequences.**
(DOC)

Table S2 **Distribution of MCs Phenotype According to Peritoneal Transport Rate.**
(DOCX)

## References

[1] Krediet RT, Zweers MM, van der Wal AC, Struijk DG (2000) Neoangiogenesis in the peritoneal membrane. Perit Dial Int 20 Suppl 2S19–25. 10911638

[2] Aroeira LS, Aguilera A, Sanchez-Tomero JA, Bajo MA, del Peso G, et al (2007) Epithelial to mesenchymal transition and peritoneal membrane failure in peritoneal dialysis patients: pathologic significance and potential therapeutic interventions. J Am Soc Nephrol 18: 2004–2013. 17568021 10.1681/ASN.2006111292

[3] Margetts PJ, Bonniaud P (2003) Basic mechanisms and clinical implications of peritoneal fibrosis. Perit Dial Int 23: 530–541. 14703193

[4] Yanez-Mo M, Lara-Pezzi E, Selgas R, Ramírez-Huesca M, Dominguez-Jimenez C, et al (2003) Peritoneal dialysis and epithelial-to-mesenchymal transition of mesothelial cells. N Engl J Med 348: 403–413. 12556543 10.1056/NEJMoa020809

[5] Loureiro J, Aguilera A, Selgas R, Sandoval P, Albar-Vizcaíno P, et al (2011) Blocking TGF-beta1 protects the peritoneal membrane from dialysate-induced damage. J Am Soc Nephrol 22: 1682–1695. 21742730 10.1681/ASN.2010111197 PMC3171939

[6] Thiery JP, Sleeman JP (2006) Complex networks orchestrate epithelial-mesenchymal transitions. Nat Rev Mol Cell Biol 7: 131–142. 16493418 10.1038/nrm1835

[7] Thiery JP, Acloque H, Huang RY, Nieto MA (2009) Epithelial-mesenchymal transitions in development and disease. Cell 139: 871–890. 19945376 10.1016/j.cell.2009.11.007

[8] Aroeira LS, Lara-Pezzi E, Loureiro J, Aguilera A, Ramírez-Huesca M, et al (2009) Cyclooxygenase-2 mediates dialysate-induced alterations of the peritoneal membrane. J Am Soc Nephrol 20: 582–592. 19158357 10.1681/ASN.2008020211 PMC2653689

[9] Ha H, Cha MK, Choi HN, Lee HB (2002) Effects of peritoneal dialysis solutions on the secretion of growth factors and extracellular matrix proteins by human peritoneal mesothelial cells. Perit Dial Int 22: 171–177. 11990400

[10] Selgas R, del Peso G, Bajo MA, Castro MA, Molina S, et al (2000) Spontaneous VEGF production by cultured peritoneal mesothelial cells from patients on peritoneal dialysis. Perit Dial Int 20: 798–801. 11216582

[11] Aroeira LS, Aguilera A, Selgas R, Ramírez-Huesca M, Pérez-Lozano ML, et al (2005) Mesenchymal conversion of mesothelial cells as a mechanism responsible for high solute transport rate in peritoneal dialysis: role of vascular endothelial growth factor. Am J Kidney Dis 46: 938–948. 16253736 10.1053/j.ajkd.2005.08.011

[12] Sandoval P, Loureiro J, González-Mateo G, Pérez-Lozano ML, Maldonado-Rodríguez A, et al (2010) PPAR-gamma agonist rosiglitazone protects peritoneal membrane from dialysis fluid-induced damage. Lab Invest 90: 1517–1532. 20531289 10.1038/labinvest.2010.111

[13] Ferrara N (1999) Vascular endothelial growth factor: molecular and biological aspects. Curr Top Microbiol Immunol 237: 1–30. 9893343 10.1007/978-3-642-59953-8_1

[14] Carmeliet P, Tessier-Lavigne M (2005) Common mechanisms of nerve and blood vessel wiring. Nature 436: 193–200. 16015319 10.1038/nature03875

[15] Cross MJ, Dixelius J, Matsumoto T, Claesson-Welsh L (2003) VEGF-receptor signal transduction. Trends Biochem Sci 28: 488–494. 13678960 10.1016/S0968-0004(03)00193-2

[16] Ferrara N, Gerber HP, LeCouter J (2003) The biology of VEGF and its receptors. Nat Med 9: 669–676. 12778165 10.1038/nm0603-669

[17] Gu C, Limberg BJ, Whitaker GB, Perman B, Leahy DJ, et al (2002) Characterization of neuropilin-1 structural features that confer binding to semaphorin 3A and vascular endothelial growth factor 165. J Biol Chem 277: 18069–18076. 11886873 10.1074/jbc.M201681200

[18] Cai H, Reed, R R (1999) Cloning and characterization of neuropilin-1-interacting protein: a PSD-95/Dlg/ZO-1 domain-containing protein that interacts with the cytoplasmic domain of neuropilin-1. J Neurosci 19: 6519–6527. 10414980 10.1523/JNEUROSCI.19-15-06519.1999 PMC6782790

[19] He Z, Tessier-Lavigne M (1997) Neuropilin is a receptor for the axonal chemorepellent Semaphorin III. Cell 90: 739–751. 9288753 10.1016/s0092-8674(00)80534-6

[20] Kolodkin AL, Levengood DV, Rowe EG, Tai YT, Giger RJ, et al (1997) Neuropilin is a semaphorin III receptor. Cell 90: 753–762. 9288754 10.1016/s0092-8674(00)80535-8

[21] Soker S, Takashima S, Miao HQ, Neufeld G, Klagsbrun M (1998) Neuropilin-1 is expressed by endothelial and tumor cells as an isoform-specific receptor for vascular endothelial growth factor. Cell 92: 735–745. 9529250 10.1016/s0092-8674(00)81402-6

[22] Bachelder RE, Wendt MA, Mercurio AM (2002) Vascular endothelial growth factor promotes breast carcinoma invasion in an autocrine manner by regulating the chemokine receptor CXCR4. Cancer Res 62: 7203–7206. 12499259

[23] Wang L, Zeng H, Wang P, Soker S, Mukhopadhyay D (2003) Neuropilin-1-mediated vascular permeability factor/vascular endothelial growth factor-dependent endothelial cell migration. J Biol Chem 278: 48848–48860. 14514674 10.1074/jbc.M310047200

[24] Li M, Yang H, Chai H, Fisher WE, Wang X, et al (2004) Pancreatic carcinoma cells express neuropilins and vascular endothelial growth factor, but not vascular endothelial growth factor receptors. Cancer 101: 2341–2350. 15476280 10.1002/cncr.20634

[25] Murga M, Fernandez-Capetillo O, Tosato G (2005) Neuropilin-1 regulates attachment in human endothelial cells independently of vascular endothelial growth factor receptor-2. Blood 105: 1992–1999. 15522955 10.1182/blood-2004-07-2598

[26] Cariboni A, Davidson K, Dozio E, Memi F, Schwarz Q, et al (2011) VEGF signalling controls GnRH neuron survival via NRP1 independently of KDR and blood vessels. Development 138: 3723–3733. 21828096 10.1242/dev.063362 PMC3152927

[27] Pan Q, Chanthery Y, Liang WC, Stawicki S, Mak J, et al (2007) Blocking neuropilin-1 function has an additive effect with anti-VEGF to inhibit tumor growth. Cancer Cell 11: 53–67. 17222790 10.1016/j.ccr.2006.10.018

[28] Appleton BA, Wu P, Maloney J, Yin J, Liang WC, et al (2007) Structural studies of neuropilin/antibody complexes provide insights into semaphorin and VEGF binding. EMBO J 26: 4902–4912. 17989695 10.1038/sj.emboj.7601906 PMC2099469

[29] Rossignol M, Gagnon ML, Klagsbrun M (2000) Genomic organization of human neuropilin-1 and neuropilin-2 genes: identification and distribution of splice variants and soluble isoforms. Genomics 70: 211–222. 11112349 10.1006/geno.2000.6381

[30] Narazaki M, Tosato G (2006) Ligand-induced internalization selects use of common receptor neuropilin-1 by VEGF165 and semaphorin3A. Blood 107: 3892–3901. 16424390 10.1182/blood-2005-10-4113 PMC1895286

[31] Matsushita A, Gotze T, Korc M (2007) Hepatocyte growth factor-mediated cell invasion in pancreatic cancer cells is dependent on neuropilin-1. Cancer Res 67: 10309–10316. 17974973 10.1158/0008-5472.CAN-07-3256

[32] West DC, Rees CG, Duchesne L, Patey SJ, Terry CJ, et al (2005) Interactions of multiple heparin binding growth factors with neuropilin-1 and potentiation of the activity of fibroblast growth factor-2. J Biol Chem 280: 13457–13464. 15695515 10.1074/jbc.M410924200

[33] Glinka Y, Prud'homme GJ (2008) Neuropilin-1 is a receptor for transforming growth factor beta-1, activates its latent form, and promotes regulatory T cell activity. J Leukoc Biol 84: 302–310. 18436584 10.1189/jlb.0208090 PMC2504713

[34] Glinka Y, Stoilova S, Mohammed N, Prud'homme GJ (2011) Neuropilin-1 exerts co-receptor function for TGF-beta-1 on the membrane of cancer cells and enhances responses to both latent and active TGF-beta. Carcinogenesis 32: 613–621. 21186301 10.1093/carcin/bgq281

[35] Tordjman R, Lepelletier Y, Lemarchandel V, Cambot M, Gaulard P, et al (2002) A neuronal receptor, neuropilin-1, is essential for the initiation of the primary immune response. Nat Immunol 3: 477–482. 11953749 10.1038/ni789

[36] Yamada Y, Oike Y, Ogawa H, Ito Y, Fujisawa H, et al (2003) Neuropilin-1 on hematopoietic cells as a source of vascular development. Blood 101: 1801–1809. 12406894 10.1182/blood-2002-01-0119

[37] Catalano A, Caprari P, Rodilossi S, Betta P, Castellucci M, et al (2004) Cross-talk between vascular endothelial growth factor and semaphorin-3A pathway in the regulation of normal and malignant mesothelial cell proliferation. Faseb J 18: 358–360. 14656993 10.1096/fj.03-0513fje

[38] Liu W, Parikh AA, Stoeltzing O, Fan F, McCarty MF, et al (2005) Upregulation of neuropilin-1 by basic fibroblast growth factor enhances vascular smooth muscle cell migration in response to VEGF. Cytokine 32: 206–212. 16289960 10.1016/j.cyto.2005.09.009

[39] Kurschat P, Bielenberg D, Rossignol-Tallandier M, Stahl A, Klagsbrun M (2006) Neuron restrictive silencer factor NRSF/REST is a transcriptional repressor of neuropilin-1 and diminishes the ability of semaphorin 3A to inhibit keratinocyte migration. J Biol Chem 281: 2721–2729. 16330548 10.1074/jbc.M507860200

[40] Latil A, Bieche I, Pesche S, Valeri A, Fournier G, et al (2000) VEGF overexpression in clinically localized prostate tumors and neuropilin-1 overexpression in metastatic forms. Int J Cancer 89: 167–171. 10754495 10.1002/(sici)1097-0215(20000320)89:2<167::aid-ijc11>3.0.co;2-9

[41] Parikh AA, Fan F, Liu WB, Ahmad SA, Stoeltzing O, et al (2004) Neuropilin-1 in human colon cancer: expression, regulation, and role in induction of angiogenesis. Am J Pathol 164: 2139–2151. 15161648 10.1016/S0002-9440(10)63772-8 PMC1615754

[42] Hansel DE, Wilentz RE, Yeo CJ, Schulick RD, Montgomery E, et al (2004) Expression of neuropilin-1 in high-grade dysplasia, invasive cancer, and metastases of the human gastrointestinal tract. Am J Surg Pathol 28: 347–356. 15104297 10.1097/00000478-200403000-00007

[43] Barr MP, Byrne AM, Duffy AM, Condron CM, Devocelle M, et al (2005) A peptide corresponding to the neuropilin-1-binding site on VEGF(165) induces apoptosis of neuropilin-1-expressing breast tumour cells. Br J Cancer 92: 328–333. 15655556 10.1038/sj.bjc.6602308 PMC2361857

[44] Baba T, Kariya M, Higuchi T, Mandai M, Matsumura N, et al (2007) Neuropilin-1 promotes unlimited growth of ovarian cancer by evading contact inhibition. Gynecol Oncol 105: 703–711. 17376520 10.1016/j.ygyno.2007.02.005

[45] Fukasawa M, Matsushita A, Korc M (2007) Neuropilin-1 interacts with integrin beta1 and modulates pancreatic cancer cell growth, survival and invasion. Cancer Biol Ther 6: 1173–1180. 17726369 10.4161/cbt.6.8.4363

[46] Pallaoro A, Braun GB, Moskovits M (2011) Quantitative ratiometric discrimination between noncancerous and cancerous prostate cells based on neuropilin-1 overexpression. Proc Natl Acad Sci U S A 108: 16559–16564. 21930955 10.1073/pnas.1109490108 PMC3189017

[47] Berge M, Allanic D, Bonnin P, de Montrion C, Richard J, et al (2011) Neuropilin-1 is upregulated in hepatocellular carcinoma and contributes to tumour growth and vascular remodelling. J Hepatol 55: 866–875. 21338642 10.1016/j.jhep.2011.01.033

[48] Jubb AM, Strickland LA, Liu SD, Mak J, Schmidt M, et al (2012) Neuropilin-1 expression in cancer and development. J Pathol 226: 50–60. 22025255 10.1002/path.2989

[49] Liang WC, Dennis MS, Stawicki S, Chanthery Y, Pan Q, et al (2007) Function blocking antibodies to neuropilin-1 generated from a designed human synthetic antibody phage library. J Mol Biol 366: 815–829. 17196977 10.1016/j.jmb.2006.11.021

[50] Bagri A, Tessier-Lavigne M, Watts RJ (2009) Neuropilins in tumor biology. Clin Cancer Res 15: 1860–1864. 19240167 10.1158/1078-0432.CCR-08-0563

[51] Haixia D, Jingsong Z, Lei J, Hairong D, Jun W, et al (2011) Gene expression of neuropilin-1 and its receptors, VEGF/Semaphorin 3a, in normal and cancer cells. Cell Biochem Biophys 59: 39–47. 20711684 10.1007/s12013-010-9109-9

[52] Bajo MA, Pérez-Lozano ML, Albar-Vizcaíno P, del Peso G, Castro MJ, et al (2011) Low-GDP peritoneal dialysis fluid ('balance') has less impact in vitro and ex vivo on epithelial-to-mesenchymal transition (EMT) of mesothelial cells than a standard fluid. Nephrol Dial Transplant 26: 282–291. 20571097 10.1093/ndt/gfq357

[53] Fernandez-Perpen A, Pérez-Lozano ML, Bajo MA, Albar-Vizcaíno P, Correa PS, et al (2012) Influence of Bicarbonate/Low-GDP Peritoneal Dialysis Fluid (Bicavera) on In Vitro and Ex Vivo Epithelial-to-Mesenchymal Transition of Mesothelial Cells. Perit Dial Int 32: 292–304. 22215656 10.3747/pdi.2010.00315 PMC3525443

[54] Selgas R, Fernández-Reyes MJ, Bosque E, Bajo MA, Borrego F, et al (1994) Functional longevity of the human peritoneum: how long is continuous peritoneal dialysis possible? Results of a prospective medium long-term study. Am J Kidney Dis 23: 64–73. 8285200 10.1016/s0272-6386(12)80814-6

[55] Ho-dac-Pannekeet MM, Atasever B, Struijk DG, Krediet RT (1997) Analysis of ultrafiltration failure in peritoneal dialysis patients by means of standard peritoneal permeability analysis. Perit Dial Int 17: 144–150. 9159834

[56] López-Cabrera M, Aguilera A, Aroeira LS, Ramírez-Huesca M, Pérez-Lozano ML, et al (2006) Ex vivo analysis of dialysis effluent-derived mesothelial cells as an approach to unveiling the mechanism of peritoneal membrane failure. Perit Dial Int 26: 26–34. 16538870

[57] Stylianou E, Jenner LA, Davies M, Coles GA, Williams JD (1990) Isolation, culture and characterization of human peritoneal mesothelial cells. Kidney Int 37: 1563–1570. 2362409 10.1038/ki.1990.150

[58] Strippoli R, Benedicto I, Pérez Lozano ML, Cerezo A, López-Cabrera M, et al (2008) Epithelial-to-mesenchymal transition of peritoneal mesothelial cells is regulated by an ERK/NF-kappaB/Snail1 pathway. Dis Model Mech 1: 264–274. 19093035 10.1242/dmm.001321 PMC2590814

[59] Strippoli R, Benedicto I, Foronda M, Perez-Lozano ML, Sanchez-Perales S, et al (2010) p38 maintains E-cadherin expression by modulating TAK1-NF-kappa B during epithelial-to-mesenchymal transition. J Cell Sci 123: 4321–4331. 21098640 10.1242/jcs.071647

[60] Lai KN, Lai KB, Lam CW, Chan TM, Li FK, et al (2000) Changes of cytokine profiles during peritonitis in patients on continuous ambulatory peritoneal dialysis. Am J Kidney Dis 35: 644–652. 10739785 10.1016/s0272-6386(00)70011-4

[61] Yang WS, Kim BS, Lee SK, Park JS, Kim SB (1999) Interleukin-1beta stimulates the production of extracellular matrix in cultured human peritoneal mesothelial cells. Perit Dial Int 19: 211–220. 10433157

[62] Zweers MM, de Waart DR, Smit W, Struijk DG, Krediet RT (1999) Growth factors VEGF and TGF-beta1 in peritoneal dialysis. J Lab Clin Med 134: 124–132. 10444025 10.1016/s0022-2143(99)90116-6

[63] Bagnard D, Vaillant C, Khuth ST, Dufay N, Lohrum M, et al (2001) Semaphorin 3A-vascular endothelial growth factor-165 balance mediates migration and apoptosis of neural progenitor cells by the recruitment of shared receptor. J Neurosci 21: 3332–3341. 11331362 10.1523/JNEUROSCI.21-10-03332.2001 PMC6762465

[64] Del Peso G, Jimenez-Heffernan JA, Bajo MA, Aroeira LS, Aguilera A, et al.. (2008) Epithelial-to-mesenchymal transition of mesothelial cells is an early event during peritoneal dialysis and is associated with high peritoneal transport. Kidney Int Suppl: S26–33. 10.1038/sj.ki.5002598 18379544

[65] Mizutani M, Ito Y, Mizuno M, Nishimura H, Suzuki Y, et al (2010) Connective tissue growth factor (CTGF/CCN2) is increased in peritoneal dialysis patients with high peritoneal solute transport rate. Am J Physiol Renal Physiol 298: F721–733. 20015945 10.1152/ajprenal.00368.2009

[66] Williams JD, Craig KJ, Topley N, Von Ruhland C, Fallon M, et al (2002) Morphologic changes in the peritoneal membrane of patients with renal disease. J Am Soc Nephrol 13: 470–479. 11805177 10.1681/ASN.V132470

[67] Numata M, Nakayama M, Nimura S, Kawakami M, Lindholm B, et al (2003) Association between an increased surface area of peritoneal microvessels and a high peritoneal solute transport rate. Perit Dial Int 23: 116–122. 12713076

[68] Pecoits-Filho R, Araujo MR, Lindholm B, Stenvinkel P, Abensur H, et al (2002) Plasma and dialysate IL-6 and VEGF concentrations are associated with high peritoneal solute transport rate. Nephrol Dial Transplant 17: 1480–1486. 12147798 10.1093/ndt/17.8.1480

[69] Boulanger E, Grossin N, Wautier MP, Taamma R, Wautier JL (2007) Mesothelial RAGE activation by AGEs enhances VEGF release and potentiates capillary tube formation. Kidney Int 71(2): 126–133. 17149374 10.1038/sj.ki.5002016

[70] Paruchuri S, Yang JH, Aikawa E, Melero-Martin JM, Khan ZA, et al (2006) Human pulmonary valve progenitor cells exhibit endothelial/mesenchymal plasticity in response to vascular endothelial growth factor-A and transforming growth factor-beta2. Circulation research 99(8): 861–869. 16973908 10.1161/01.RES.0000245188.41002.2c PMC2810464

[71] Medici D, Shore EM, Lounev VY, Kaplan FS, Kalluri R, et al. Conversion of vascular endothelial cells into multipotent stem-like cells. Nature medicine 16(12): 1400–1406. 10.1038/nm.2252 PMC3209716 21102460

[72] Vega S, Morales AV, Ocana OH, Valdes F, Fabregat I, et al (2004) Snail blocks the cell cycle and confers resistance to cell death. Genes Dev 18: 1131–1143. 15155580 10.1101/gad.294104 PMC415638

[73] Yu MA, Shin KS, Kim JH, Kim YI, Chung SS, et al (2009) HGF and BMP-7 ameliorate high glucose-induced epithelial-to-mesenchymal transition of peritoneal mesothelium. J Am Soc Nephrol 20: 567–581. 19193779 10.1681/ASN.2008040424 PMC2653690

[74] Loureiro J, Schilte M, Aguilera A, Albar-Vizcaíno P, Ramírez-Huesca M, et al (2010) BMP-7 blocks mesenchymal conversion of mesothelial cells and prevents peritoneal damage induced by dialysis fluid exposure. Nephrol Dial Transplant 25: 1098–1108. 20067910 10.1093/ndt/gfp618

[75] Kihm LP, Muller-Krebs S, Klein J, Ehrlich G, Mertes L, et al. Benfotiamine protects against peritoneal and kidney damage in peritoneal dialysis. J Am Soc Nephrol 22: 914–926. 21511829 10.1681/ASN.2010070750 PMC3083313

