# Supplementary figures and images for: Functional Relevance of the Switch of VEGF Receptors/Co-Receptors during Peritoneal Dialysis-Induced Mesothelial to Mesenchymal Transition
Source: PLoS One. 2013 Apr 9;8(4):e60776. doi: 10.1371/journal.pone.0060776 (PMC3621952; doi:10.1371/journal.pone.0060776)

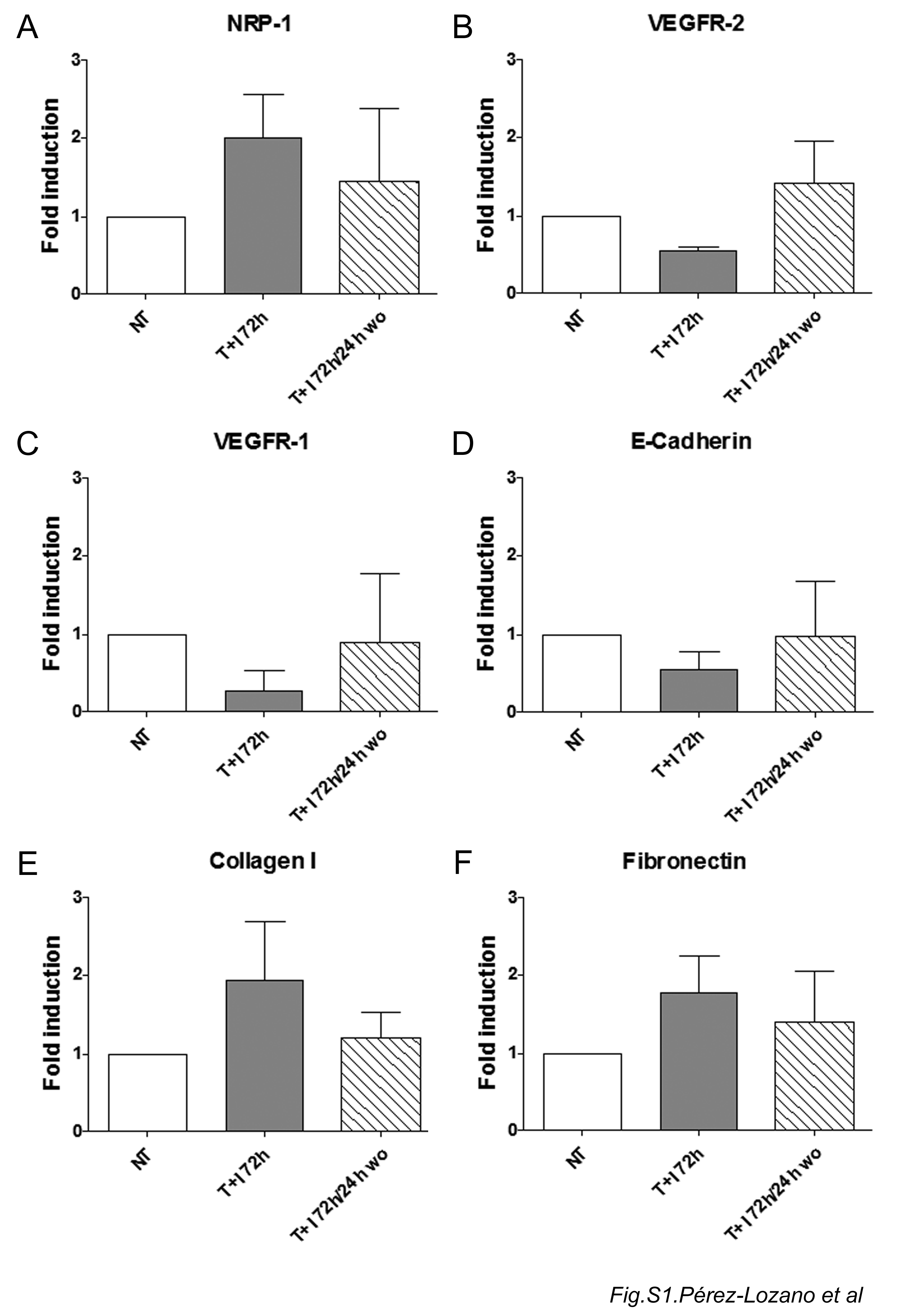

Supplement: Figure S1 — Effect on VEGF receptors and co-receptors after TGF-β1 and IL-1β removal. (A–C) mRNA levels of VEGF receptors/co-receptors were analyzed by quantitative RT-PCR. The results represent the fold induction of mRNA expression of VEGF receptors in omentum-derived MCs treated with TGF-β1 plus IL-1 β (T+I 72 h) and omentum-derived MCs after T+I withdrawal (T+I 72 h/24 h wo) compared with untreated MCs (NT). (D–F) mRNA levels of mesenchymal markers were analyzed by quantitative RT-PCR. Histograms represent the expression of E-cadherin, collagen I and fibronectin after the treatment with T+I (T+I 72 h) and after T+I withdrawal (T+I 72 h/24 h wo) compared to non treated omentum-derived MCs (NT). (TIF) [file pone.0060776.s001.tif]

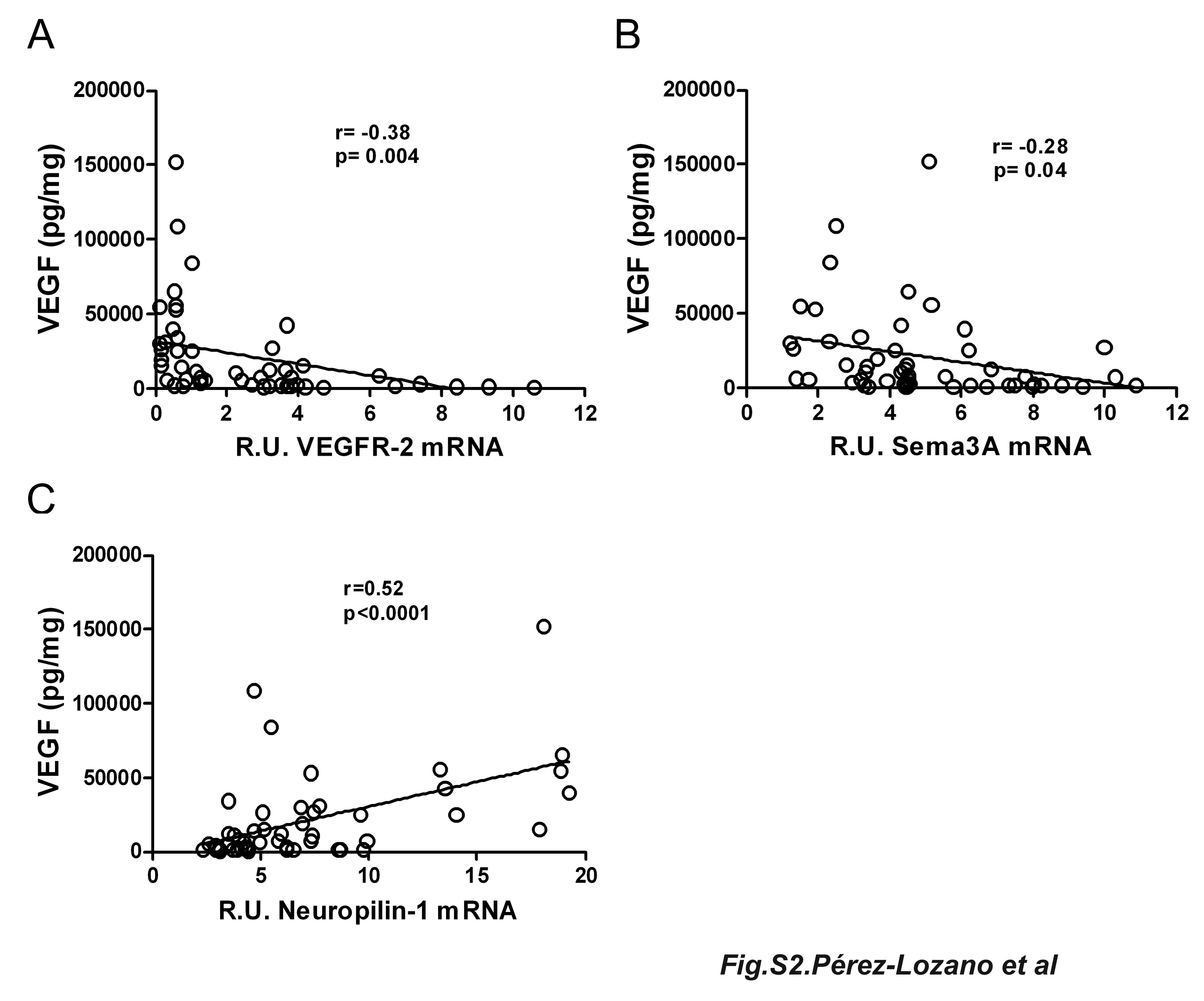

Supplement: Figure S2 — Correlations between secreted levels of VEGF and mRNA levels in effluent-derived MCs. (A) Negative correlation between VEGF levels and VEGFR-2 mRNA expression levels (p = 0.004). (B) Negative correlation between VEGF levels and Sema-3A mRNA expression (p = 0.04). (C) Positive correlation between secreted VEGF and Nrp1 mRNA expression levels (p<0.0001). Data are depicted as mean value ± SE. Symbols show statistical differences between groups. (TIF) [file pone.0060776.s002.tif]
