# Supplementary material for: Functional Relevance of the Switch of VEGF Receptors/Co-Receptors during Peritoneal Dialysis-Induced Mesothelial to Mesenchymal Transition
Source: PLoS One. 2013 Apr 9;8(4):e60776. doi: 10.1371/journal.pone.0060776 (PMC3621952; doi:10.1371/journal.pone.0060776)
Supplement: Table S1 — Oligonucleotides Sequences. (DOC) [file pone.0060776.s003.doc]

**Table S1. Oligonucleotides Sequences**

|  | | **Primers Sequences for Real-Time PCR** | |  |
| --- | --- | --- | --- | --- |
| Gene | Gen Bank No. | Forward primers | Reverse primers | |
| VEGFR-1  Tm=64ºC | NM_002019 | 5´CAAGTGGCCAGAGGCATGGAGTT 3´ | 5´GATGTAGTCTTTACCATCCTG 3´ | |
| VEGFR-2  Tm=62ºC | AF063658 | 5´TGAGCATGGAAGAGGATTCTG 3´ | 5´CTCTTTCGCTTACTGTTCTGC 3´ | |
| VEGFR-3  Tm=66ºC | NM_002020 | 5´TCTGCCTGGGACTCCTGGAC 3´ | 5´ACCGGTGTCGATGACGTGTG 3´ | |
| Nrp-1  Tm=68ºC | BT006995 | 5´AAGGTTTCTCAGCAAACTACAGTG 3´ | 5´GGGAAGAAGCTGTGATCTGGTC 3´ | |
| Nrp-2  Tm=62ºC | NM_018534 | 5´TTCAAGACACCAAGTGAGAGG 3´ | 5´ACAATCTTCTGGTTGGGTTCG 3´ | |
| Sema-3A  Tm=58ºC | NM_006080 | 5´TGCCAAGGCTGAAATTATCC 3´ | 5´CCTACTCCGTTCCTCATCCA 3´ | |
| Snail-1  Tm=55ºC | NM_005985.3 | 5´GCAAATACTGCAACAAGG 3´ | 5´GCACTGGTACTTCTTGACA 3´ | |
| E-Cadh  Tm=62ºC | NM_004360 | 5´TGAAGGTGACAGAGCCTCTG 3´ | 5´TGGGTGAATTCGGGCTTGTT 3´ | |
| N-Cadh  Tm=55ºC | M34064.1 | 5´GAAAACCCTTATTTTGCCCC 3´ | 5CACAGGATCTATTTTTAGCC 3 | |
| Col-I  Tm=64ºC | NM_000088.3 | 5´ GCTATGATGAGAAATCAACCG 3´ | 5´ GCTTCCCCATCATCTCCATTC 3´ | |
| Fn  Tm=66ºC | AB191261 | 5´ CCTGAAGCTGAAGAGACTTGC 3´ | 5´ CGTTTCTCCGACCACATAGGA 3´ | |
| H3  Tm= 62ºC | M11353 | 5´AAAGCCGCTCGCAAGAGTGCG 3´ | 5´ACTTGCCTCCTGCAAAGCAC 3´ | |
