# Supplementary material for: Functional Relevance of the Switch of VEGF Receptors/Co-Receptors during Peritoneal Dialysis-Induced Mesothelial to Mesenchymal Transition
Source: PLoS One. 2013 Apr 9;8(4):e60776. doi: 10.1371/journal.pone.0060776 (PMC3621952; doi:10.1371/journal.pone.0060776)
Supplement: Table S2 — Distribution of MCs Phenotype According to Peritoneal Transport Rate. (DOCX) [file pone.0060776.s004.docx]

**Table S2. Distribution of MCs Phenotype According to Peritoneal Transport Rate.**

|  | **Cr-MTC <11 ml/min** | **Cr-MTC ≥ 11 ml/min** | **Total** |
| --- | --- | --- | --- |
| **Epithelioid** | 21 | 9 | 30 |
| **Non-epithelioid** | 8 | 13 | 21 |
| **Total** | 29 | 22 | 51 |

**Note**: Statistic differences in distribution of epithelioid and non-epithelioid phenotype in relation to peritoneal transport (2-tail Fisher exact test, p=0.043).
